# Metabolomics and *In-Silico* Analysis Reveal Critical Energy Deregulations in Animal Models of Parkinson’s Disease

**DOI:** 10.1371/journal.pone.0069146

**Published:** 2013-07-23

**Authors:** Pierre O. Poliquin, Jingkui Chen, Mathieu Cloutier, Louis-Éric Trudeau, Mario Jolicoeur

**Affiliations:** 1 Department of Chemical Engineering, École Polytechnique de Montréal, Montréal, Quebec, Canada; 2 GERAD and Department of Chemical Engineering, École Polytechnique de Montréal, Montréal, Quebec, Canada; 3 Department of Pharmacology, Faculty of Medicine, Université de Montréal, Montréal, Quebec, Canada; National Institutes of Health, United States of America

## Abstract

Parkinson’s disease (PD) is a multifactorial disease known to result from a variety of factors. Although age is the principal risk factor, other etiological mechanisms have been identified, including gene mutations and exposure to toxins. Deregulation of energy metabolism, mostly through the loss of complex I efficiency, is involved in disease progression in both the genetic and sporadic forms of the disease. In this study, we investigated energy deregulation in the cerebral tissue of animal models (genetic and toxin induced) of PD using an approach that combines metabolomics and mathematical modelling. In a first step, quantitative measurements of energy-related metabolites in mouse brain slices revealed most affected pathways. A genetic model of PD, the Park2 knockout, was compared to the effect of CCCP, a complex I blocker. Model simulated and experimental results revealed a significant and sustained decrease in ATP after CCCP exposure, but not in the genetic mice model. In support to data analysis, a mathematical model of the relevant metabolic pathways was developed and calibrated onto experimental data. In this work, we show that a short-term stress response in nucleotide scavenging is most probably induced by the toxin exposure. In turn, the robustness of energy-related pathways in the model explains how genetic perturbations, at least in young animals, are not sufficient to induce significant changes at the metabolite level.

## Introduction

Parkinson’s disease (PD) is a pernicious neurodegenerative disease for which no cure exists. Two important cellular hallmarks of this disease are the formation of cellular inclusions named Lewy Bodies, and the degeneration of dopamine-containing neurons of the ventral midbrain, mainly within the *substantia nigra pars compacta* [1], [2]. Lewy bodies appear to be the result of a degenerative metabolic process implicating the aggregation of alpha-synuclein protein, and the failure of energy-intensive biochemical mechanisms such as disposal of damaged proteins [3]. Since the disease arises from different causes: environmental toxicity, genetic mutations and brain aging, general disease prevalence can hardly be defined [4]. However, fundamentally to all biological systems, metabolic energy homeostasis is critical, especially in cells that consume energy at high rates, such as brain neurons.

Toxins used in farming, such as paraquat (herbicide), rotenone (pesticide) and maneb (fungicide) are known to induce specific PD-related symptoms in animal models [5]. These toxins inhibit mitochondrial complexes 1 (paraquat and rotenone) and 3 (maneb) involved in cellular respiration. Also, solvents such as toluene and n-hexane, and carbon disulfide used in solvents and pesticides, have been shown to cause neuronal death by mitochondrial damage [6]. MPTP (1-methyl-4-phenyl-1,2,3,6-tetrahydropyridine), inadvertently obtained in the incomplete synthesis of the recreational drug MPPP (4′-methyl-α-pyrrolidinopropiophenone), leads to rapid appearance of PD symptoms within few days [7]–[9]. Similarly to other ionophores, MPTP uncouples cellular respiration and leads to a complete shutdown of mitochondrial function and of other cellular processes. Genetic mutations related to PD have been found in seven different genes: PARK1, 3, 5, 8 (dominant) and PARK2, 6, 7 (recessive). These genes encode proteins involved in neurotransmission (PARK1), protein quality control and cellular stress responses (PARK2, PARK5, and PARK7), regulation of mitochondrial function (PARK6) and in the regulation of the cyclosqueleton and protein-protein interactions (PARK8) [10]. Even though familial forms of PD are not frequent (at most 20% of all PD cases), elucidation of their molecular mechanisms could help to identify causes of more common idiopathic forms of the disease [11]–[13]. Interestingly, a final common pathway of many PD gene defects appears to be mitochondrial dysfunction with perturbations of cellular energy production. In addition to the above-mentioned factors, cellular dysfunctions linking PD to aging are not completely identified, however it is known that the latter involves reduced availability of metabolic energy and impaired clearance of damaged proteins and cellular by-products. Therefore, it is likely that this third factor is also linked to impaired regulation of cellular energy [14].

In this work, we have studied the effect of PD-related perturbations on brain cells metabolism. Metabolites were measured in brain tissue from Parkin KO (knockout) mice, as well as in brain tissue exposed to the complex I antagonist CCCP (*carbonyl cyanide m-chloro phenyl hydrazone*). The genetic model of Parkinson’s disease selected for the present study was Parkin KO mice that present mitochondrial efficiency reduction. This gene is situated on locus 6q25.2–q27 [15] and codes for a E3-ubiquitin protein ligase [16] involved in the degradation of damaged proteins through the Ubiquitin Proteasome System (UPS) [17]. Parkin is also involved with Pink1 in mitophagy, a quality control mechanism removing damaged mitochondria [18]. Impaired mitochondrial and protein degradation may lead to protein aggregation and perturbed cellular energetics. In the present study, animals were produced by mating heterozygote mice to obtain KO and wild-type (WT) littermates. Because a number of toxins such as MPTP, and PD gene mutations including those in the Parkin gene, perturb mitochondrial function and in particular the complex I, we compared tissues obtained from Parkin KO mice to WT tissues treated with CCCP. This ionophore is known to dissipate the pH gradient across the mitochondrial membrane, leading to the loss of ATP production, an energetic shuttle critical for cellular metabolism [19].

As expected, energy production pathways are extremely robust because of multiple feedback interactions, which in turn, induce emergent properties such as homeostasis [20]. These regulatory properties are likely to be extremely important in how the brain reacts to the energetic perturbations/stresses occurring in PD. However, it is extremely tedious to investigate this issue with experimental data alone [21]. In that context, a dynamic modelling platform can efficiently complement experimental studies [22], [23]. To evaluate the relative implications of measured *ex-vivo* data, an *in-silico* platform was developed and model simulations were used to rationally integrate the experimental dataset and then allow studying mice brain cells metabolomic state behaviour. From model simulations, we attempted to model the impact of parkin gene KO and complex I blockade on energetic cell metabolism. Therefore, in order to assess the dynamics of metabolic events involved in this phenomenon, we developed a kinetic-metabolic model describing concentrations in nutrients and cell metabolites, as well as metabolic fluxes with time, in brain tissue. The current study thus presents a kinetic-metabolic model of central metabolism and energy metabolic pathways describing the dynamics of energy related metabolites after PD inducing perturbations.

## Materials and Methods

The protocols that require evaluation have been approved by the “Ethics committee of animal experimentation” of the Université de Montréal.

### Slice Preparation

Mouse brain slices were prepared and rapidly transferred in Petri dishes. Two slices were evaluated each 10 min (for a total of eight time points) and homogenised in an alcohol extractor to arrest cellular metabolism. In two experiments, a control group (wild type mouse) was compared to either the genetic model or the toxin-induced model. Mice were anaesthetized with halothane and immediately killed by decapitation. The brain was rapidly removed and placed in ice-cold carboxygenated (95% O_2_ and 5% CO_2_) cutting-solution (glycerol-containing artificial cerebrospinal fluid (G-ACSF)) containing (in mM): NaCl, 125 mM; KCl, 2.5 mM; KH_2_PO_4_, 0.3 mM; NaHCO_3_, 26 mM; CaCl_2_, 2.4; GLC, 10 mM; MgSO_4_, 1.3 mM; at 300 mOsm. The olfactory bulbs and cerebellum were removed and the rest of the brain sliced using a vibrating microtome (Leica VT1000S®) in order to make 300- µm thick coronal slices. During slice preparation, the tissue was always maintained immersed in 4°C ACSF. Each slice was delicately transferred to a Petri dish continuously perfused with oxygenated ACSF. After preparation, brain slices from both mice were allowed to recover for about 30 min.

### Stress and Control Groups

Two brains (a control and a test sample) were sectioned and processed in parallel for each experiment. Individual slices were processed at a rate of one per 10 min for a total of about 75 min. Parkin KO mice on a C57bl/6 background were used as a genetic model of PD, and their oxidative phosphorylation capacity was taken to be 90% of that of the wild-type (WT) [17]. Wild-type littermates were used a control. A subset of slices from WT mice were exposed to 10 µM CCCP to inhibit complex I function. The inhibitor was added in the experimental group after the second sampling, 15 min following the first extraction. After the collection of two samples exposed to CCCP, remaining experimental slices were transferred to a Petri dish containing normal ACSF, as for the control group.

### Preparation of Intracellular Extracts

The understanding of cells metabolic activity in response to perturbations requires the simultaneous evaluation of many compounds involved in the cellular metabolic network. Several extraction methods for metabolite analysis have been reported, and based on previously reported results [24]–[26] and our own studies, a modified procedure using cold methanol was found to be the most appropriate since it combines mild extraction strength conditions and high enzymatic inactivation.

The two slices selected for each extract were transferred using a brush from a Petri dish to a centrifuge tube, which was then centrifuged at 21 000 *g* at 4°C for one min; the ACSF supernatant was then removed using a flame-pulled Pasteur pipette. The pre-weighted centrifuge vial was then reweighted to deduct the collected fresh brain mass and 200 µL of an 80% v/v mixture of methanol and water, cooled on dry ice, was added. The tissue was then homogenized using a motorised pillar, vortexed and sonicated to disrupt cell membranes, precipitate proteins and arrest metabolic reactions. An additional 200 µL of methanol/water was next added and the homogenization, vortexing and sonication steps were repeated. The samples were maintained at −80°C for further analyses.

The sample was resuspended and vortexed after addition of 0.2 g of sand to improve homogenisation, and micro-centrifuged at 21 000 *g* for 7 min at 4°C. The resulting supernatant was collected and considered as the first extract. After the addition of 0.2 mL of ice-cold 50% v/v methanol/water to the pellet, the sample was again vortexed, cooled in an ice-water bath and sonicated for three rounds. The sample was then micro-centrifuged at 21 000 *g* and 4°C for 5 min to produce the second extract; the latter was added to the first. Next, 0.2 mL of ice-cold water was added to the pellet and the vial was vortexed and micro-centrifuged at 21 000 *g* and 4°C for 3 min to give the third extract. Finally, the vial contained the pooled extracts was micro-centrifuged at 21 000 *g* and 4°C for 5 min. The supernatant was then filtered in a syringe with a 0.2 µm filter (Millipore) and stored at −80°C until LCMS analysis.

### Sample Analysis

The samples were thawed and vortex mixed before analysis. Samples were kept at 4°C during LCMS autosampling. Minimal LCMS autosampling loading was performed since it has been reported that metabolites degrade faster at higher temperatures, compared to −80°C.

Nucleotide and organic acids together with sugar phosphates quantification was performed as described in Hammami et al. [27]. Quantification of metabolites (nucleotides and organic acids) was performed by integrating peak areas and using standard linear calibration curves. Extraction specific concentrations of metabolites were calculated by normalizing the quantity of metabolites in the slice extracts to the fresh weight of the slices. When the peak area integration led to extrapolation from the linear standard calibration curve, proper dilution was performed using 50% (v/v) methanol in water and the sample reanalysed. The concentration quantification methods used in the present study refer to whole biological sample homogenisation; the various cellular compartments components and volumes are summed in global concentrations values. Based on this extraction method’s limits, the model considers an average brain slice cells composition.

### Nucleotides Quantification

Nucleotides in extracts were analyzed using a 1290 UPLC system coupled to a 6460 triple quadrupole mass spectrometer (Agilent Technologies®). Nucleotides were separated by a Symmetry C18 column (Waters®) equipped with a Security C18 guard-column (Waters®) using ion-pair method. DMHA (N,N-dimethylhexylanine, Sigma®) was used as an ion-pair reagent to improve the signal-to-noise ratio with positive ionization mode.

### Sugar Phosphates and Organic Acids Quantification

Organic acids and phosphate sugars concentrations were assessed using the aforementioned UPLC-MS/MS system using a Hypercarb® column and a Hypercarb pre-column (Thermo Fisher®).

### Extracellular Components

ACSF cellular environment samples were taken directly from the Petri dish. The four major extracellular components such as glucose, lactate, glutamine and glutamate were measured with a blood analyser (YSI 2700 Select Biochemistry Analyzer®). Two modules were used in parallel; glucose and lactate were measured simultaneously on an analyser, while another measured glutamine and glutamate simultaneously.

### Statistical Analysis

Data are shown as mean ± SEM (standard error of mean) of *n* = 3 independent experiments from brain slices prepared from three mice. Outliers were removed using box-plot analysis.

### Cell Populations

In the present study, complete brain slices were homogenized. It was therefore not possible to distinguish between metabolites originating from the different cell types (neurons, astrocytes, oligodendrocytes, microglia, etc.). Considering that cell populations are not homogeneous throughout the rostro-caudal extent of the brain, we were careful to select comparable sets of slices from control and test samples. In addition, although PD is associated with the degeneration of a limited number of brain regions including the locus ceruleus and the substantia nigra pars compacta, in the present study, brain tissue was collected from the whole brain. We did not attempt to collect only the ventral mesencephalon, where the substantia nigra is localized, as this would have lead to insufficient quantities of cells and thus of metabolites; an experimental limitation determined from the detection limit of the LCMS and UPLCMSMS equipments used. Although in these mice the Parkin gene was knocked out from all cells, it is possible that by analysing whole brains, we missed perturbations in cell metabolism that were more specific to the affected nuclei.

### The Model

**Figure 1:**
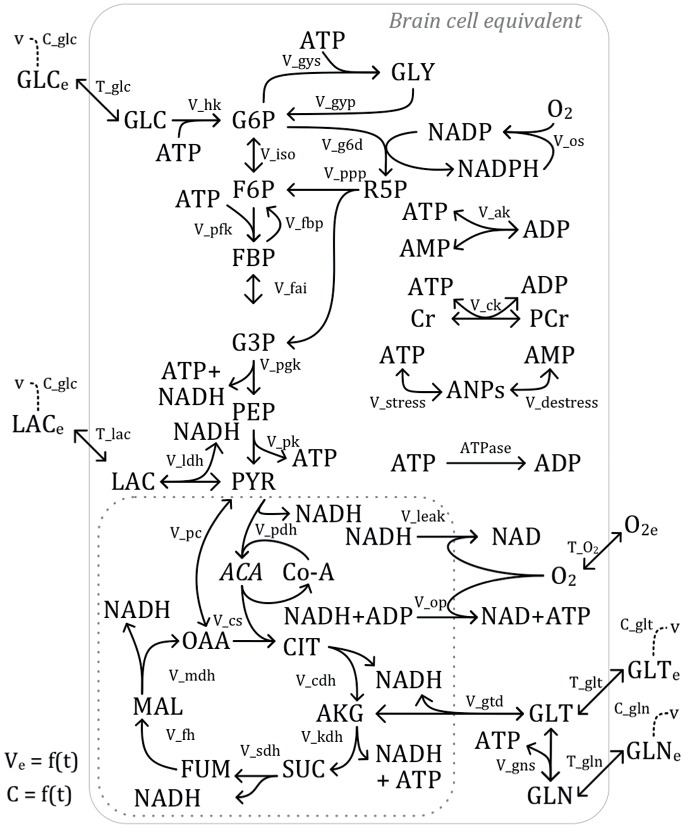
Energy metabolism model for the cerebral tissue. The states of the model (in capital letters) are defined as follows: GLC, glucose; G6P, glucose-6-phosphate; F6P, fructose-6-phosphate; FBP, fructose-biphosphate; G3P, glyceraldehyde-3-phosphate; PEP, phosphoenolpyruvate; PYR, pyruvate; GLY, glycogen; R5P, ribose-5-phosphate; Cr (PCr), creatine (phosphocreatine); LAC, lactate; ACA, acetyl-coenzyme-A; CIT, citrate; AKG, α-ketoglutarate; SUC, succinate; FUM, fumarate; MAL, malate; OAA, oxaloacetate; GLT, glutamate; GLN, glutamine; NAD (NADH), nicotniamide adenine dinucleotide (reduced); NADP (NADPH), phosphorylated nicotinamide adenine dinucleotide (reduced); ATP, adenosine-triphosphate; ADP, adenosine-diphosphate; AMP, adenosine-monophosphate; O_2_, oxygen; ANPs, non-free adenosine-“n”phosphate nucleotides; V_e_, extracellular volume; subscript “e” refers to extracellular. Reactions (*in italic*) are defined in Supplementary Materials. The intracellular volume delimited by the dotted line refers to the mitochondrial volume.

The metabolic pathways investigated in this study are presented in Figure 1. Details concerning the model (transient mass balances, parameters etc.) are provided in the Supplementary Material (SM); with the description of the kinetic-metabolic model (Table S1), fluxes’ kinetic formulation (Table S2), fluxes’ functions (Table S3), state variables and initial conditions (Table S4), maximal fluxes’ rates, affinity, inhibition, threshold, stoichiometric ratios and other constants (Table S5). All functions in Table S5 are widely used in control engineering, and a description of secondary parameters can be found in major textbooks in systems’ dynamics and control engineering. The model structure and kinetics were taken or adapted from literature [28–29; and references therein], and it was implemented in Matlab® (The Mathworks, Inc.®) using the Systems Biology Toolbox® (SBT) [30]. The model Matlab® code is provided as supplementary material. The model accounts for biochemical reactions occurring in all brain cells, mostly neurons and astrocytes, and was constructed not to represent a specific cerebral region nor cell type or function. We thus used concentrations units (µM) for the metabolites and limited the modelling to a generic set of reactions that are known to occur in these cell types. This was to ensure a physiologically realistic model that could eventually be adapted to other experimental investigations. Most of the molecules involved in the model are present in relatively large amounts implying hundreds of thousands (or more) of each molecule for a typical neuron. From a modelling perspective, this means that the overall reaction system can be meaningfully modelled within a deterministic framework to describe cells’ metabolic network and dynamics. Other PD-related modeling efforts also successfully used this approach [28]; [31]–[33].

### Pathways Considered

In this work, we have limited our scope integrating central carbon metabolism and energetic pathways. Pathways represented in the model originate from a database of combined information from literature as well as the web-database *BioCyc.org* [34]. The model considers molecular transport across the cellular membrane (identified with prefix “*T*”, as *T_glc* for the transport of GLC). As the main influx, glycolysis is described with a high level of detail. The modelling is focused on highly controlled enzymatic reactions (e.g. *V_pfk* for the flux of phosphofructokinase) and important branch point. The pentose phosphate pathway (PPP), which is the major pathway for anti-oxidative action, is represented with its two glycolytic connecting points. Even if the latter pathway is extensively simplified to a single state, the by-products stoichiometry (regeneration of 2 NADPH for each GLC cycling through PPP) is consistent with the full pathway. Pyruvate from the glycolytic pathway is either converted to lactate through reversible lactate dehydrogenase, or transferred to the mitochondria and consumed in the TCA cycle. While some intermediates (such as AKG) can be used as branch point to amino acids (GLT) consumption/production, the cycle’s main function is to perform reduction of NAD shuttles to NADH. This in turn allows ATP regeneration from ADP by oxidative phosphorylation in the mitochondria matrix. Although this pathway involves many enzymatic complexes and sub-compartments, a simple one-step reaction with parallel proton leakage is considered here.

The two major energetic buffers are considered: phosphocreatine and glycogen. Creatine kinase transfers the high energy phosphate link from ATP to Cr and the resulting molecule PCr can in turn be used to directly regenerate ATP during periods of high demand. Glycogen is considered as a single state with a reversible reaction from G6P. Although energy is used to obtain G6P, glycogen can in turn be used temporarily if GLC transport becomes limiting during periods of high-energy demand, thus creating a buffer effect between low and high energy demand periods [28].

### Consideration on States

Most model states are determined as a distinct mass balance with a rate of change equal to the difference between production and consumption fluxes. Alternatively, moiety conservation is used to reduce the number of differential equations, when possible. For example, the sum of PCr and Cr is constant and Cr can be expressed as “*Cr = PCr_TOT_ – PCr*”. In those cases, the unique assumption is that the total pool of metabolites is constant over the time frame of the experiment. This approach reduces the number of differential equations and computing time. In energy metabolism, some enzymatic activities reach an extremely rapid equilibrium and this can also be used to reduce the number of differential equations, while still describing the associated state variables with algebraic equations. As an example, even if uridine-triphosphate (UTP) is necessary for glycogen storage, the enzyme *nucleoside diphosphate kinase* covers the use immediately and stabilises the UTP pool with ATP. In the model, we thus only represent ATP as the global energy currency and its dynamics will be representative of the overall energetic state.

### Parameters Estimation

Although the model presented here could eventually be used to estimate confidence intervals on the parameters, this was not the immediate objective of the present work. Our objective was rather to develop a kinetic-metabolic model structure that can be challenged with experimental data. Along with this process of model calibration, we tested its usefulness to retrieve various fundamental informations (i.e. fluxes, parameter sensitivity etc.) that can hardly be obtained experimentally, with regards to energetic regulation during the early events of PD pathogenesis. The parameters of the model were first estimated from the available literature, as detailed below, and then calibrated minimizing the error between simulations and experimental data, as previously described [29]. The measured rate of GLC intake by the brain tissue was also used to estimate the reaction rates, when possible. Most of the enzymatic reactions are described using simple Michaelis-Menten saturation-type kinetics. Being globally accepted in the scientific community as a satisfactory mathematical abstraction for substrate-protein interactions, quantification of the apparent affinity of proteins to their different substrates is common, as shown by the large amount of work available in comprehensive shared web-databases like *brenda-enzymes.info* [35]. Understanding that a model is a simplified representation of a phenomenon, or of a network of biochemical reactions in this case, the kinetic parameters determined in this work have to be seen as global; they also include what is not described, such as metabolic regulation as well as other biochemical reactions that are taking place but that are lumped in this model. The parameter values determined in this work may thus differ from the strict real value for a biochemical reaction, but bring a global view on a group of regulated biochemical reactions occurring around each metabolite described in the model.

### K_M_ apparent Affinity

The affinity of protein-substrate interactions has not been measured for all organisms or cell types. In this work we thus gathered the most relevant values from the databases. When possible, the affinity values were taken for mouse brain proteins (see Table S5 in Supplemental material). Otherwise, the values come from mammalian cells or from an average of many different eukaryotic cells. However, these values were only used as reasonable first estimates and were later adjusted during model calibration process.

### Feedback Regulation

In addition to multi-substrate and reversible Michaelis-Menten flux expression, for specific enzymes or pathways, the mathematical description requires complementary feedback expressions to improve its robustness and fidelity with regards to commonly seen processed such as energy homeostasis. Hill inhibition kinetics on phosphofructokinase, threshold activation and inhibition of glycogen on the glycogen buffer pathway, and ADP to ATP ratio-controlled oxidative phosphorylation are examples of feedback regulation mechanisms that were implemented.

### V_M_ Maximal Flux Rates

The maximum rate of model fluxes, especially glycolytic fluxes, can be estimated from the basal (i.e. before perturbation) GLC uptake rate and LAC excretion rate, as measured from the extracellular GLC and LAC concentrations. The rate of GLC intake imposes a global limit on the glycolytic fluxes and also on its branchpoints; the sum of fluxes at a branchpoint has to balance when in the ‘baseline’ steady-state. The rate of LAC excretion indicates, indirectly, the proportion of GLC that underwent complete oxidation, which in turn is an estimate for the TCA fluxes. Thus, with known values for the K_M_ and other kinetic parameters (Table S5), if we have an estimate of the flux and measurements for all of the concentrations, the kinetic equation can be solved with V_M_ as the only unknown. This gives a realistic estimate for these parameters, at least for the ‘baseline’. Subsequently, model fine-tuning was done both manually and with computational optimisation routines included in the SBT. As presented below, the model could benefit from further optimisation for specific experimental cases. However, within the scope of this work, we considered sufficient to achieve a satisfactory overall fit with one set of parameters for all of the experimental conditions.

### Stress Implementation

Another addition was also made to the model to allow a sudden change linked to the addition of CCCP to the media (or in our case, the transfer of slices in a CCCP prepared ACSF). Because this ionophore uncouples the respiration chain as it increases proton leakage across the membrane barrier, the stress is represented in the model as an increase of the natural mitochondrial leakage process. As the proton gradient has a direct effect on the efficacy of the oxidative phosphorylation pathway, and considering that pH gradients are not represented in the model, the leakage is modeled as oxidizing directly NADH to NAD without ADP phosphorylation to ATP. As the stress was implemented as a pulse, such mathematical description is added to the model using a sigmoidal-shape function. Since the toxin diffuses easily into the brain slice, a fast acting sigmoid is imposed in the opening pulse function. Finally, since the CCCP was washed out of the slice relatively slowly, a slow acting sigmoid is added for the pulse function closing term.

## Results and Discussion

The complete model has 41 fluxes, 38 states, 82 literature constants, 28 simulation related parameters, five *in-vitro* calculated values and 46 *in-silico* defined parameters (Table S5) from model calibration step to experimental dataset. The model considers two zones, the cellular compartment with its interior volumes (cytosol with mitochondria) and the extracellular medium, which includes the extracellular matrix space. The model structure and parameters value calibration was performed using the experimental data and literature. In addition to simple weight measures, blood analysers and LCMS methods allowed for the qualification of 17 compounds, such as GLC, LAC, GLN, GLT, ATP, ADP, AMP, NADPH, PYR, MAL, SUC, FUM, AKG, G6P, R5P, F6P and PEP. Only the most significant data are presented in the following, together with *in-silico* simulations; the remaining material is provided in the Supplementary Materials.

In the following sub-sections, *ex-vivo* measured values from wet-lab experimentations are presented with corresponding *in-silico* results from computer simulations. The comparison of controlled conditions to toxin exposure and parkin KO genetic modification are presented successively.

### Exposure to Toxins Induces Severe Energy Deregulation

**Figure 2:**
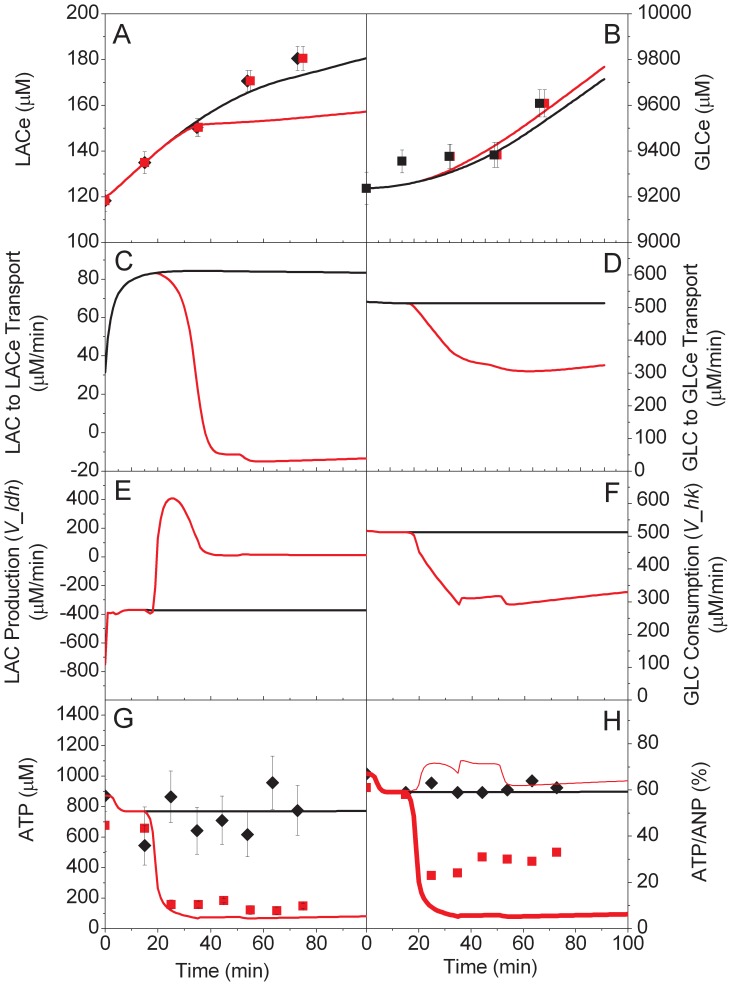
Effect of toxin exposure on energy dynamics. Experimental data of WT control (♦) and CCCP stressed (▪) brain cells, and model simulations of control (black line) and CCCP stressed (red line) cells. LACe (A), GLCe (B), LACe to LAC transport (C), GLCe to GLC transport (D), LAC production (V_ldh) (E), GLC consumption (V_hk) (F), ATP (G), ATP-to-(ATP+ADP+AMP) (♦,▪, black line, dotted red line) and ATP-to-(ATP+ADP+AMP+ANPs) (thick red line) ratios (H).

In the wild-type tissue case (Figure 2), the model simulation was in accordance with extracellular measurements. This indicates that the overall consumption/production rates in the model are in accordance with the experimental system.

The observed increase in GLC (+3% over 75 min) is not due to GLC excretion from the slices. The Petri dish being open to unsaturated air and sparged with dry gases, significant evaporation is likely to have happened during the experiment. Effective metabolites flows were then corrected by estimated evaporation measurements and analysis of simulated fluxes; results showed that the effect of evaporation was slightly stronger than GLC consumption by the slices and thus had to be considered.

**Figure 3:**
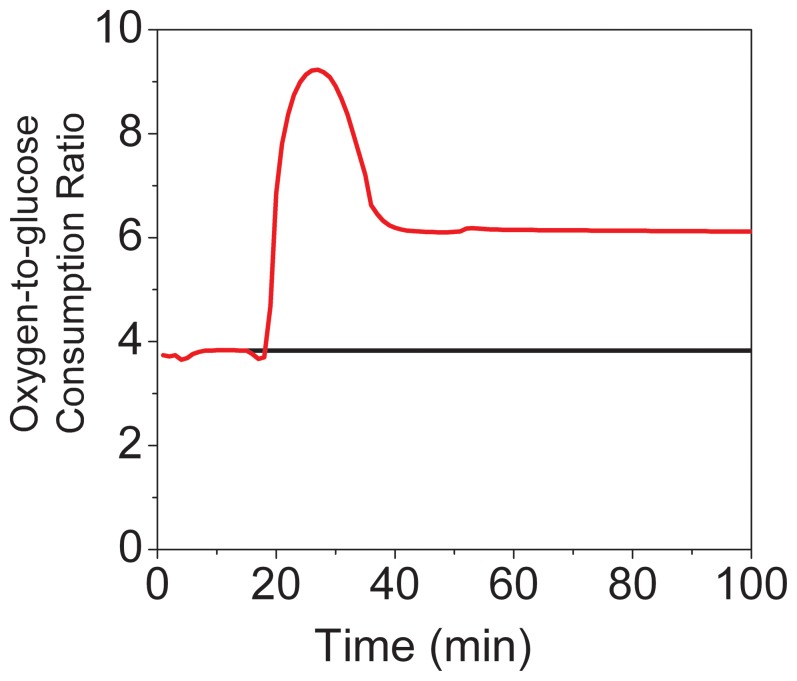
Basal indicator for WT and CCCP stressed models. Model simulation of control (black line) and CCCP stressed (red line) brain cells oxygen-to-glucose-consumption ratio.

On the other hand, in the case of LAC, the increase was much more significant (+50% in 75 min) and most of the increase is likely to have arisen from LAC excretion from the slices. This indicates an imbalance between glycolysis and oxidative phosphorylation, which is coherent with previous work on brain physiology. Furthermore, the basal unstressed consumption ratio of oxygen-to-glucose indicator, as calculated by equation 1 and presented in Figure 3, is within the 3.5 to 5.5 range observed physiologically [36]. This shows that the slicing process and diffusion within the slice did not seem to affect the overall metabolic rates. A severe diffusional problem would most likely have resulted in a lower O_2_-to-GLC consumption ratio.
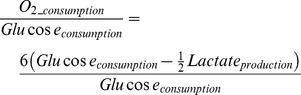
(1)


Results show that ATP profile for the wild-type tissue did not exhibit any clear trend, and that experimental and measurement noise may dominates. In the model, a consistent result (i.e. no trend) is achieved because the negative feedback on various steps of energy metabolism produces a homeostatic response in terms of ATP. More specifically, hexokinase, phospho-fructokinase and pyruvate kinase enzymes are activated when ATP is low, and oxidative phosphorylation is inhibited when ATP is high (refer to section 3.2 and reactions 26 (*V_hk*), 36 (*V_pfk*), 38 (*V_pk*) and the ADP-on-ATP ratio as considered in reaction 32 (*V_op*) in Table S2).

The short-term dynamic response during and following exposure to CCCP reveals a rapid and significant drop of ATP concentration that remains until the end of the experimental period. This dynamic response is reproduced in the model by different means. The uncoupling of oxidative phosphorylation by the CCCP causes the initial drop in energy. In this new state of low energy, the metabolic regulation for nucleotides scavenging is initiated [36] as described in equations 5 and 41, respectively from Table S1 and Table S2. As the recovery from this stress (nucleotide regeneration) is slow (reaction 16 in Table S2), the system remains in a low energetic state for the duration of the experiment.

Although experimental observations are on a relatively short timescale, it is clear that the exposure to environmental toxins such as CCCP can induce rapid and permanent changes of energy metabolism. In our experimental system, ATP levels after exposure to CCCP are reduced to 25% of their initial levels without causing immediate tissue death. The latter result comes from visual observation of the slices and also from the measured changes in GLC and LAC, showing sustained metabolic activity after exposure. This indicates a clear metabolic robustness even after strong perturbations. It is possible that exposure to CCCP-like toxins such as MPTP (as observed in humans) could be as severe, with a significant drop in ATP, but without causing immediate cell death. In the longer term (days or weeks), the lack of complete metabolic recovery would however certainly lead to necrosis and loss of dopaminergic neurons, which agree with the literature on MPTP usage [7].

The only discrepancy between the modelling and experimental data is the slower LAC increase in the simulation with CCCP (Fig. 2-A). This suggests that the model with decoupled energy production tends to re-uptake LACe contributing to re-balance TCA. The principal cause for this TCA unbalancing is that the model considers the NADH as a simple proton gradient. This leads to strong reduction of NADH instead of proton leak and further over availability of NAD. As a consequence, NADH production by all NAD single co-substrate consuming enzymes such as PYR dehydrogenase (*V_pdh*) and CIT dehydrogenase (*V_cdh*) from TCA cycle are up-regulated. Hence, the global unbalancing of the TCA cycle could be described in a more complete way if all connected pathways were considered. However, based on the measurements of extracellular lactate, its rate of production after 60 min is not significant. Thus the model is in accordance with this observation as shown in Figure 2.E.

Furthermore, the reduction of NADPH allows such a “turbo” mode for the overall metabolism as seen in Figure 3, where the oxygen-to-glucose consumption ratio indicator increases to a high value of 6 after a higher (∼9) transient response. Although stable for the considered timescale, such an operating mode would eventually lead to higher risk of damage by oxidative stress and loss of extracellular LAC.

### Energy Robustness under Parkin KO Genetic Perturbation

**Figure 4:**
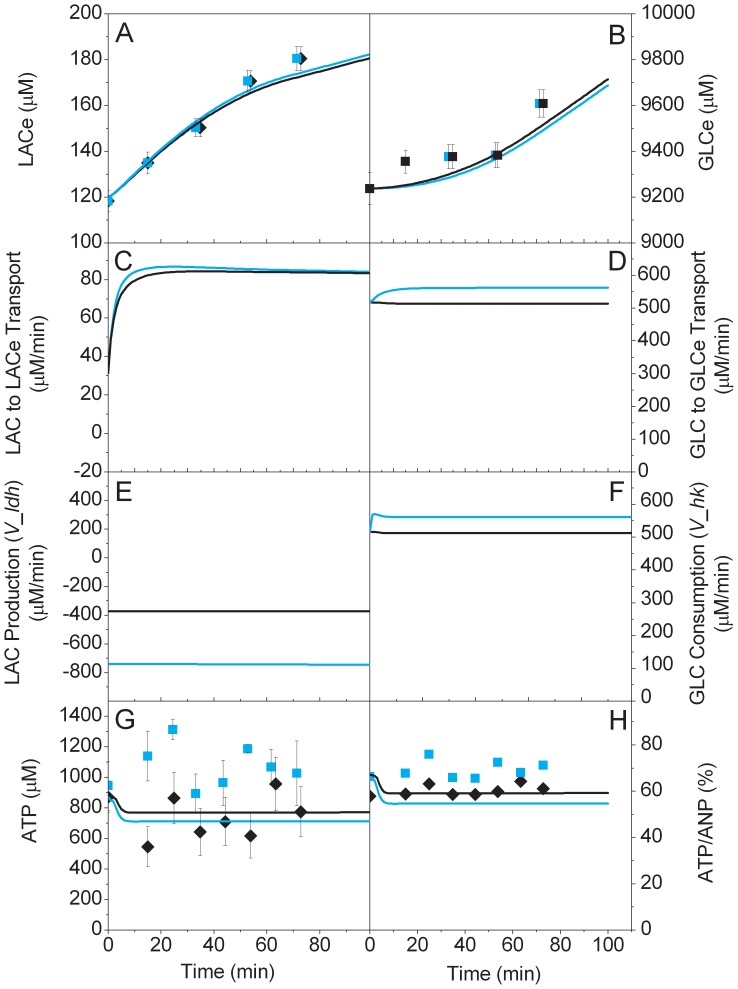
Effect of parkin gene knockout on brain cells energy dynamics. Experimental data of WT control (♦) and parkin knockout (▪) brain cells, and model simulations of control (black line) and parkin knockout (bleue line) cells. LACe (A), GLCe (B), LACe to LAC transport (C), GLCe to GLC transport (D), LAC production (V_ldh) (E), GLC consumption (V_hk) (F), ATP (G), ATP-to-(ATP+ADP+AMP) ratio (H).

Model simulations are in agreement with extracellular measurements of cell energetics markers (Figure 4). In the same way as for the control WT case, the KO model has overall consumption/production rates that agree with that of the experimental system.

**Figure 5:**
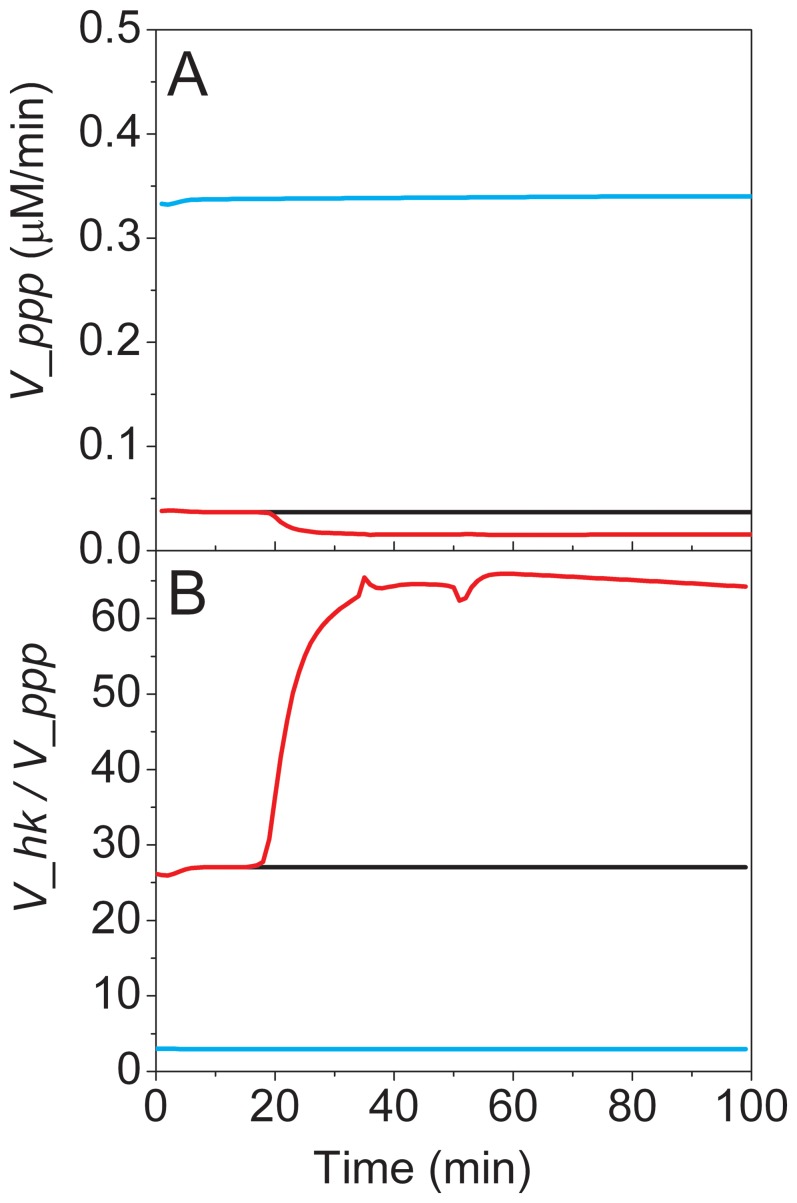
Comparison of fluxes and metabolic ratios. Model simulations of WT control (black line), CCCP stressed (red line) and parkin knockout (bleue line) brain cells. Characteristic pentose phosphate pathway flux rate (V_ppp) (A), Glucose consumption rate (V_hk) –to- characteristic pentose phosphate pathway flux rate (V_ppp) (B).

Although all profiles are generally similar to the genetically unaltered model, a slight adaptation can be observed. While lactate production is still positive, as the flux of lactate dehydrogenase (*V_ldh*) is negative, the reaction rate is approximately doubled. This result is probably a metabolic adaptation following genetic modification. In fact, the deletion of the *parkin gene* is likely leading to the accumulation of a number of damaged proteins, such as misfolded alpha-synuclein, which could lead to increased oxidative stress, as described by Cloutier and Wellstead [37]. The most affected pathway of the model for this genetically induced oxidative stress is the pentose phosphate pathway, since its important cofactor NADPH allows regeneration (reduction) of GSSH to GSH of the glutathione oxidative stress reducing pathway (with glutathione reductase) [38]. Although the magnitude of the oxidative stress generation is multiplied by ten in this case, cells’ energetic regulation seems to compensate for such strong perturbation. The ratio of pentose phosphate over glycolysis, with time, (Figure 5) reveals a variation of the genetically stressed mouse model compared to both control and CCCP stressed mice models, where simple energy regulation leads to a global system adaptation at another possible operating point.

The use of the model thus allows detailed description of the fluxes, invisible from an experimental level. In this case, tissue from the KO mice exhibits identical molecular concentrations, but increased reaction rates allowing a sustainable metabolism.

### Conclusion

A mathematical model for brain energy metabolism in the context of PD related stresses has been developed and validated using experimental data. The major observation from this work is that environmental exposure to a toxin such as the complex I inhibitor CCCP is a severe stress in terms of deregulation of energy metabolism and that a response is initiated when ATP levels are irreversibly reduced. In the case of the genetic defect examined (parkin gene KO), the modelling, supported by experimental evidences, suggests that the biochemical regulation of energy metabolism increases metabolic flows without affecting the metabolite concentrations in order to allow the system to adapt to a certain level of stress. Although the experiments and simulations are on a relatively short timescale, the results suggest how energy can be rapidly deregulated by strong environmental stresses relevant to PD. In the long term, a certain level of adaptation could certainly occur, but over time, some damage (i.e. protein misfolding or oxidative stress) would accumulate, leading to cellular disorganisation and cell death [37]. Other integrative modelling efforts also show that irreversible effects could be rapidly induced in PD [39], which is coherent with the rapid onset of neuronal dysfunction observed in humans exposed to MPTP [40].

## Supporting Information

Table S1 ***In silico*** **model mass balances.**
(DOCX)

Table S2 **Fluxes kinetics description.**
(DOCX)

Table S3 **Fluxes functions description.**
(DOCX)

Table S4 **State variables and initial conditions.**
(DOCX)

Table S5 **Maximal fluxes rates, affinity, inhibition, threshold, stoichiometric ratios and other constants.**
(DOCX)

Table S6 **Model Matlab® code.**
(DOCX)
