# Correction: Metabolomics and *In-Silico* Analysis Reveal Critical Energy Deregulations in Animal Models of Parkinson’s Disease

**DOI:** 10.1371/journal.pone.0112009

**Published:** 2014-10-27

**Authors:** The PLOS ONE Staff

There are errors in the article. Please see below for a complete list of the errors and their corrections.

In the sixth sentence of the Abstract, “a complex I blocker” should be “a mitochondrial uncoupler.”

In the second sentence of the third paragraph of the Introduction, “complex I antagonist CCCP” should be “mitochondrial uncoupler CCCP.”

There is an error in the fifth sentence of the “Stress and Control” section of Materials and Methods. The correct sentence is: A subset of slices from WT mice were exposed to 10 µM CCCP to inhibit mitochondrial function.

There is an error in the second sentence of the Conclusion. The correct sentence is: The major observation from this work is that environmental exposure to the toxic mitochondrial uncoupler CCCP is a severe stress in terms of deregulation of energy metabolism and that a response is initiated when ATP levels are irreversibly reduced.
