# Supplementary material for: Metabolomics and In-Silico Analysis Reveal Critical Energy Deregulations in Animal Models of Parkinson’s Disease
Source: PLoS One. 2013 Jul 23;8(7):e69146. doi: 10.1371/journal.pone.0069146 (PMC3720533; doi:10.1371/journal.pone.0069146)
Supplement: Table S1 — In silico model mass balances. (DOCX) [file pone.0069146.s001.docx]

**Supplementary material**

**Table S1:** *In silico* model mass balances

| ODE | State description |
| --- | --- |
| 1 | $\frac{d\left( ACA \right)}{dt}= +V_{pdh}-V_{cs}$ |
| 2 | $\frac{d\left( AKG \right)}{dt}= +V_{cdh}-V_{kdh}+V_{gtd}$ |
| 3 | $\frac{d\left( AMP \right)}{dt}= -V_{ak}+V_{destress}$ |
| 4 | $\frac{d\left( ANPs \right)}{dt}= +V_{stress}-V_{destress}$ |
| 5 | $\frac{d\left( ATP \right)}{dt}= -V_{hk}-V_{pfk}+V_{pgk}+V_{pk}-V_{pc}+V_{kdh}-V_{gns}-V_{gys}-V_{ck}+Eta_{op}*n_{op_{atp}}*V_{op}-V_{atpase}-V_{ak}-V_{stress}$ |
| 6 | $\frac{d\left( CIT \right)}{dt}= +V_{cs}-V_{cdh}$ |
| 7 | $\frac{d\left( F6P \right)}{dt}= +V_{iso}-V_{pfk}+n_{ppp_{f6p}}*V_{ppp}+V_{fbp}$ |
| 8 | $\frac{d\left( FBP \right)}{dt}= +V_{pfk}-V_{fai}-V_{fbp}$ |
| 9 | $\frac{d\left( FUM \right)}{dt}= +V_{sdh}-V_{fh}$ |
| 10 | $\frac{d\left( G3P \right)}{dt}= +n_{fai_{g3p}}*V_{fai}-V_{pgk}+V_{ppp}$ |
| 11 | $\frac{d\left( G6P \right)}{dt}= +V_{hk}-V_{iso}-n_{ppp_{g3p}}*V_{g6d}-V_{gys}+V_{gyp}$ |
| 12 | $\frac{d\left( GLC \right)}{dt}= +T_{glc}-V_{hk}$ |
| 13 | $\frac{d\left( GLCe \right)}{dt}= -R_{volume}* T_{glc}+C_{glc}$ |
| 14 | $\frac{d\left( GLN \right)}{dt}= -T_{gln}+V_{gns}$ |
| 15 | $\frac{d\left( GLNe \right)}{dt}= +R_{volume}* T_{gln}+C_{gln}$ |
| 16 | $\frac{d\left( GLT \right)}{dt}= -T_{glt}-V_{gtd}-V_{gns}$ |
| 16 | $\frac{d\left( GLTe \right)}{dt}= +R_{volume}* T_{glt}+C_{glt}$ |
| 17 | $\frac{d\left( GLY \right)}{dt}= +V_{gys}-V_{gyp}$ |
| 18 | $\frac{d\left( LAC \right)}{dt}= -T_{lac}-V_{ldh}$ |
| 19 | $\frac{d\left( LACe \right)}{dt}= +R_{volume}* T_{lac}+C_{lac}$ |
| 20 | $\frac{d\left( MAL \right)}{dt}= +V_{fh}-V_{mdh}$ |
|  |  |
|  | **Table S1:** *In silico* model mass balances (continued) |
|  |  |
| 21 | $\frac{d\left( NADH \right)}{dt}= +V_{pgk}+V_{ldh}+V_{pdh}+V_{cdh}+V_{kdh}+n_{sdh_{nadh}}*V_{sdh}+V_{mdh}+V_{gtd}-V_{leak}-V_{op}$ |
| 22 | $\frac{d\left( NADPH \right)}{dt}= +n_{g6d_{nadph}}*V_{g6d}-V_{os}$ |
| 23 | $\frac{d\left( O2 \right)}{dt}= +T_{o2}-n_{op_{o2}}*V_{op}-n_{leak_{o2}}*V_{leak}$ |
| 24 | $\frac{d\left( O2e \right)}{dt}= +T_{o2e}$ |
| 25 | $\frac{d\left( OAA \right)}{dt}= +V_{mdh}-V_{cs}+V_{pc}$ |
| 26 | $\frac{d\left( PCr \right)}{dt}= +V_{ck}$ |
| 27 | $\frac{d\left( PEP \right)}{dt}= +V_{pgk}-V_{pk}$ |
| 28 | $\frac{d\left( PYR \right)}{dt}= +V_{pk}-V_{pdh}-V_{pc}+V_{ldh}$ |
| 29 | $\frac{d\left( R5P \right)}{dt}= +V_{g6d}-V_{ppp}$ |
| 30 | $\frac{d\left( SUC \right)}{dt}= +V_{kdh}-V_{sdh}$ |
| 31 | $\frac{d\left( V \right)}{dt}= -V_{evap}$ |
