# Supplementary material for: Metabolomics and In-Silico Analysis Reveal Critical Energy Deregulations in Animal Models of Parkinson’s Disease
Source: PLoS One. 2013 Jul 23;8(7):e69146. doi: 10.1371/journal.pone.0069146 (PMC3720533; doi:10.1371/journal.pone.0069146)
Supplement: Table S2 — Fluxes kinetics description. (DOCX) [file pone.0069146.s002.docx]

**Supplementary material**

**Table S2:** Fluxes kinetics description

| Flux | Description |
| --- | --- |
| *1* | $C_{glc}= EVAP\left( K_{evap},GLCe,V \right)$ |
| *2* | $C_{gln}= EVAP\left( K_{evap},GLNe,V \right)$ |
| *3* | $C_{glt}= EVAP\left( K_{evap},GLTe,V \right)$ |
| *4* | $C_{lac}= EVAP\left( K_{evap},LACe,V \right)$ |
| *5* | $T_{glc}= TMM\left( Vm_{t_{glc}},Km_{t_{glc}},GLCe,GLC \right)$ |
| *6* | $T_{gln}= TMM\left( Vm_{t_{gln}},Km_{t_{gln}},GLN,GLNe \right)$ |
| *7* | $T_{glt}= TMM\left( Vm_{t_{glt}},Km_{t_{glt}},GLT,GLTe \right)$ |
| *8* | $T_{lac}= TMM\left( Vm_{t_{lac}},Km_{t_{lac}},LAC,LACe \right)$ |
| *9* | $T_{o2}= K_{t_{o2}}* DIFF\left( O2e,O2 \right)$ |
| *10* | $T_{o2e}= 1 - PULSE\left( A_{p_{o2}},time,T_{p_{on_{o2}}},F_{p_{on_{o2}}},P_{p_{on_{o2}}},T_{p_{off_{o2}}},F_{p_{off_{o2}}},P_{p_{off_{o2}}} \right)$ |
| *11* | $V_{ak}= RMM\left( \begin{aligned} Vm_{ak_{f}},Km_{ak_{atp}},ATP,Km_{ak_{amp}},AMP,0,1, \\ Vm_{ak_{r}},Km_{ak_{adp}},ADP,0,1,0,1 \end{aligned} \right)$ |
| *12* | $V_{atpase}= Vm_{atpase}*MM\left( Km_{atpase_{atp}},ATP \right)* PULSE\left( \begin{aligned} A_{p_{atpase}},time,T_{p_{on_{atpase}}},F_{p_{on_{atpase}}},P_{p_{on_{atpase}}}, \\ T_{p_{off_{atpase}}},F_{p_{off_{atpase}}},P_{p_{off_{atpase}}} \end{aligned} \right)$ |
| *13* | $V_{cdh}= Vm_{cdh}*MM\left( Km_{cdh_{cit}},CIT \right)* MM\left( Km_{cdh_{nad}},NAD \right)$ |
| *14* | $V_{ck}= RMM\left( \begin{aligned} Vm_{ck_{f}},Km_{ck_{cr}},Cr,Km_{ck_{atp}},ATP,0,1, \\ Vm_{ck_{r}},Km_{ck_{pcr}},PCr,Km_{ck_{adp}},ADP,0,1 \end{aligned} \right)$ |
| *15* | $V_{cs}= Vm_{cs}*MM\left( Km_{cs_{aca}},ACA \right)* MM\left( Km_{cs_{oaa}},OAA \right)$ |
| *16* | $V_{destress}= Vm_{destress}*MM\left( Km_{destress_{anps}},ANPs \right)$ |
| *17* | $V_{evap}= K_{evap}$ |
| *18* | $V_{fai}= RMM\left( Vm_{fai_{f}},Km_{fai_{fbp}},FBP,0,1,0,1,Vm_{fai_{r}},Km_{fai_{g3p}},G3P,0,1,0,1 \right)$ |
| *19* | $V_{fbp}= Vm_{fbp}*MM\left( Km_{fbp_{fbp}},FBP \right)$ |
| *20* | $V_{fh}= Vm_{fh}*MM\left( Km_{fh_{fum}},FUM \right)$ |
| *21* | $V_{g6d}= Vm_{g6d}*MM\left( Km_{g6d_{g6p}},G6P \right)* MM\left( Km_{g6d_{nadp}},NADP \right)$ |
|  | **Table S2:** Fluxes kinetics description (continued) |
|  |  |
| *22* | $V_{gns}= RMM\left( Vm_{gns_{f}},Km_{gns_{glt}},GLT,0,1,0,1,Vm_{gns_{r}},Km_{gns_{gln}},GLN,0,1,0,1 \right)$ |
| *23* | $V_{gtd}= RMM\left( \begin{aligned} Vm_{gtd_{f}},Km_{gtd_{glt}},GLT,Km_{gtd_{nad}},NAD,0,1, \\ Vm_{gtd_{r}},Km_{gtd_{akg}},AKG,Km_{gtd_{nadh}},NADH,0,1 \end{aligned} \right)$ |
| *24* | $V_{gyp}= Vm_{gyp}*MM\left( Km_{gyp_{gly}},GLY \right)$ |
| *25* | $V_{gys}= Vm_{gys}*MM\left( Km_{gys_{g6p}},G6P \right)* MM\left( Km_{gys_{atp}},ATP \right)* SWITCH_{I}\left( GLY,TRH_{i_{gys_{gly}}},F_{i_{gys_{gly}}} \right)$ |
| *26* | $V_{hk}= Vm_{hk}*MM\left( Km_{hk_{glc}},GLC \right)* MM\left( Km_{hk_{atp}},ATP \right)* SWITCH_{I}\left( G6P,TRH_{i_{hk_{g6p}}},F_{i_{hk_{g6p}}} \right)$ |
| *27* | $V_{iso}= RMM\left( Vm_{iso_{f}},Km_{iso_{g6p}},G6P,0,1,0,1,Vm_{iso_{r}},Km_{iso_{f6p}},F6P,0,1,0,1 \right)$ |
| *28* | $V_{kdh}= Vm_{kdh}*MM\left( Km_{kdh_{akg}},AKG \right)* MM\left( Km_{kdh_{adp}},ADP \right)* MM\left( Km_{kdh_{nad}},NAD \right)$ |
| *29* | $V_{ldh}= RMM\left( \begin{aligned} Vm_{ldh_{f}},Km_{ldh_{lac}},LAC,Km_{ldh_{nad}},NAD,0,1, \\ Vm_{ldh_{r}},Km_{ldh_{pyr}},PYR,Km_{ldh_{nadh}},NADH,0,1 \end{aligned} \right)$ |
| *30* | $V_{leak}= Vm_{leak}*MM\left( Km_{leak_{nadh}},NADH \right)* PULSE\left( A_{p_{leak}},time,T_{p_{on_{leak}}},F_{p_{on_{leak}}},P_{p_{on_{leak}}},T_{p_{off_{leak}}},F_{p_{off_{leak}}},P_{p_{off_{leak}}} \right)$ |
| *31* | $V_{mdh}= Vm_{mdh}*MM\left( Km_{mdh_{mal}},MAL \right)* MM\left( Km_{mdh_{nad}},NAD \right)$ |
| *32* | $V_{op}= Vm_{op}*MM\left( Km_{op_{nadh}},NADH \right)* MM\left( Km_{op_{adp}},ADP \right)* MM\left( Km_{op_{o2}},O2 \right)* RATIO\left( ADP,ATP \right)$ |
| *33* | $V_{os}= V_{op}*\left( 1-Eta_{op} \right)* PULSE\left( A_{p_{os}},time,T_{p_{on_{os}}},F_{p_{on_{os}}},P_{p_{on_{os}}},T_{p_{off_{os}}},F_{p_{off_{os}}},P_{p_{off_{os}}} \right)$ |
| *34* | $V_{pc}= Vm_{pc}*MM\left( Km_{pc_{pyr}},PYR \right)* MM\left( Km_{pc_{atp}},ATP \right)$ |
| *35* | $V_{pdh}= Vm_{pdh}*MM\left( Km_{pdh_{pyr}},PYR \right)* MM\left( Km_{pdh_{nad}},NAD \right)* MM\left( Km_{pdh_{coa}},CoA \right)$ |
|  |  |
|  | **Table S2:** Fluxes kinetics description (continued) |
|  |  |
| *36* | $V_{pfk}= Vm_{pfk}*MM\left( Km_{pfk_{f6p}},F6P \right)* MM\left( Km_{pfk_{atp}},ATP \right)* HILL\left( Ki_{pfk_{atp}},ATP,nH_{pfk} \right)$ |
| *37* | $V_{pgk}= Vm_{pgk}*MM\left( Km_{pgk_{g3p}},G3P \right)* MM\left( Km_{pgk_{adp}},ADP \right)$ |
| *38* | $V_{pk}= Vm_{pk}*MM\left( Km_{pk_{pep}},PEP \right)* MM\left( Km_{pk_{adp}},ADP \right)$ |
| *39* | $V_{ppp}= Vm_{ppp}*MM\left( Km_{ppp_{r5p}},R5P \right)$ |
| *40* | $V_{sdh}= Vm_{sdh}*MM\left( Km_{sdh_{suc}},SUC \right)* MM\left( Km_{sdh_{nad}},NAD \right)$ |
| *41* | $V_{stress}= Vm_{stress}*MM\left( Km_{stress_{atp}},ATP \right)* \left( 1 - SWITCH_{I}\left( ATP,TRH_{i_{stress_{atp}}},F_{i_{stress_{atp}}} \right) \right)* \left( -1 + PULSE\left( \begin{aligned} A_{p_{leak}},time,T_{p_{on_{leak}}},F_{p_{on_{leak}}},P_{p_{on_{leak}}}, \\ T_{p_{off_{leak}}},F_{p_{off_{leak}}},P_{p_{off_{leak}}} \end{aligned} \right) \right)$ |
