# Supplementary material for: Metabolomics and In-Silico Analysis Reveal Critical Energy Deregulations in Animal Models of Parkinson’s Disease
Source: PLoS One. 2013 Jul 23;8(7):e69146. doi: 10.1371/journal.pone.0069146 (PMC3720533; doi:10.1371/journal.pone.0069146)
Supplement: Table S3 — Fluxes functions description. (DOCX) [file pone.0069146.s003.docx]

**Supplementary material**

**Table S3:** Fluxes functions description

| Function | Description* |
| --- | --- |
| *1* | $MM\left( K,S \right)= \left( \frac{S}{K + S} \right)$ |
| *2* | $TMM\left( Vm,Km,Si,Se \right)= \left( Vm*\left( \frac{Si}{Si + Km}-\frac{Se}{Se + Km} \right) \right)$ |
| *3* | $RMM\left( \begin{aligned} Vmf,Kmf1,Sf1,Kmf2,Sf2,Kmf3,Sf3, \\ Vmr,Kmr1,Sr1,Kmr2,Sr2,Kmr3,Sr3 \end{aligned} \right)= \left( \begin{aligned} Vmf*\left( \frac{Sf1}{Sf1 + Kmf1} \right)*\left( \frac{Sf2}{Sf2 + Kmf2} \right)*\left( \frac{Sf3}{Sf3 + Kmf3} \right)- \\ Vmr*\left( \frac{Sr1}{Sr1 + Kmr1} \right)*\left( \frac{Sr2}{Sr2 + Kmr2} \right)*\left( \frac{Sr3}{Sr3 + Kmr3} \right) \end{aligned} \right)$ |
| *4* | $RMA\left( Kf,Sf1,Sf2,Sf3,Kr,Sr1,Sr2,Sr3 \right)=$  $( Kf*Sf1*Sf2*Sf3 - Kr*Sr1*Sr2*Sr3 )$ |
| *5* | $DIFF\left( Se,Si \right)= \left( Se - Si \right)$ |
| *6* | $PMM\left( Km,S,n \right)= \left( \frac{S^{n}}{Km^{n}+ S^{n}} \right)$ |
| *7* | $EVAP\left( K_{evap},Se,V \right)= \left( K_{evap}*\frac{Se}{V} \right)$ |
| *8* | $RATIO\left( UPPER,LOWER \right)= \left( \frac{UPPER}{LOWER} \right)$ |
| *9* | $HILL\left( Ki,S,n \right)= \left( \frac{1}{1+\left( \frac{S}{Ki} \right)^{n}} \right)$ |
| *10* | $SWITCH_{I}\left( S,St,a \right)= \left( 1-\frac{1}{1+ exp\left( -a*\left( S-St \right) \right)} \right)$ |
| *11* | $SWITCH_{A}\left( S,St,a \right)= \left( \frac{1}{1+ exp\left( -a*\left( S-St \right) \right)} \right)$ |
| *12* | $PULSE\left( A,t,t1,a1,p1,t2,a2,p2 \right)= \left( \left( 1 + unitpulseSB\left( time,t1,t2 \right)* A*\left( 1/{1+ \left( \frac{1-p1}{p1} \right)*exp\left( -a1*\left( t-t1 \right) \right)}-1/{1+ \left( \frac{1-p2}{p2} \right)*exp\left( -a2*\left( t-t2 \right) \right)} \right) \right) \right)$ |
| * Nomenclature of the acronyms used as functions  HILL Hill-type activation or inhibition kinetics  MM Michaelis-Menten kinetics  PMM Power-type Michaelis-Menten kinetics  Pulse Sigmoidal step-type perturbation kinetics  Ratio Metabolic flux ratio  RMM Reversible Michaelis-Menten kinetics  Switch Smooth increased/saturation activation or inhibition kinetics  TMM Michaelis-Menten kinetics for transmembrane transport | |
