# Supplementary material for: Metabolomics and In-Silico Analysis Reveal Critical Energy Deregulations in Animal Models of Parkinson’s Disease
Source: PLoS One. 2013 Jul 23;8(7):e69146. doi: 10.1371/journal.pone.0069146 (PMC3720533; doi:10.1371/journal.pone.0069146)
Supplement: Table S4 — State variables and initial conditions. (DOCX) [file pone.0069146.s004.docx]

**Table S4.** State variables and initial conditions

| Component | Description | Value | Units |
| --- | --- | --- | --- |
| ACA | Acetyl-CoezymeA | 74.5 | μM |
| AKG | α-Ketoglutarate | 596 | μM |
| AMP | Adenosine Monophosphate | 254 | μM |
| ANPs | Stressed Adenosine N-phosphated | 0 | μM |
| ATP | Adenosine triphosphate | 872 | μM |
| CIT | Citrate | 290 | μM |
| F6P | Fructose 6-Phosphate | 130 | μM |
| FBP | Fructose Bi-Phosphate | 18.65 | μM |
| FUM | Fumarate | 11.36 | μM |
| G3P | Glyceraldehyde 3-Phosphate | 14.89 | μM |
| G6P | Glucose 6-Phosphate | 644.95 | μM |
| GLC | Glucose | 1000 | μM |
| GLCe | Extracellular Glucose | 9237 | μM |
| GLN | Glutamine | 20 | μM |
| GLNe | Extracellular Glutamine | 4 | μM |
| GLT | Glutamate | 200 | μM |
| GLTe | Extracellular Glutamate | 18 | μM |
| GLY | Glycogen | 2635.5 | μM |
| LAC | Lactate | 400 | μM |
| LACe | Extracellular Lactate | 120 | μM |
| MAL | Malate | 596 | μM |
| NADH | Nicotinamide adenine dinucleotide (reduced) | 230 | μM |
| NADPH | Nicotinamide adenine dinucleotide phosphate (reduced) | 65.08 | μM |
| O2 | Oxygen | 1234.1 | μM |
| O2e | Extracellular Oxygen | 1235 | μM |
| OAA | Oxaloacetate | 5.39 | μM |
| PCr | PhosphoCreatine | 1826.3 | μM |
| PEP | Phosphoenolpyruvate | 6.3 | μM |
| PYR | Pyruvate | 49 | μM |
| R5P | Ribose 5-phosphate | 21.5 | μM |
| SUC | Succinate | 500 | μM |
| V | Extracellular Volume | 0.04 | L |
| ANP_T | Sum of Phosphorylated Adenosine nucleotide | 1300.76 | μM |
| CoA_T | Sum of CoA and ACA | 76.3 | μM |
| Cr_T | Sum of Creatine (standalone and phosphorylated) | 2500 | μM |
| NAD_T | Sum of Nicotinamide adenine dinucleotide (reduced and oxydized) | 1070 | μM |
| NADP_T | Sum of Nicotinamide adenine dinucleotide phosphate (reduced and oxydized) | 66 | μM |
| ADP | Adenosine Diphosphate | 174.76 | μM |
| ANP | Adenosine N-Phosphate | 1300.76 | μM |
| Cells | Tissue slice | 0.000548 | L |
| CoA | CoenxymeA | 1.8 | μM |
| Cr | Creatine | 673.7 | μM |
| NAD | Nicotinamide adenine dinucleotide (oxidized) | 840 | μM |
| NADP | Nicotinamide adenine dinucleotide phosphate (oxidized) | 0,92 | μM |
