# Supplementary material for: Metabolomics and In-Silico Analysis Reveal Critical Energy Deregulations in Animal Models of Parkinson’s Disease
Source: PLoS One. 2013 Jul 23;8(7):e69146. doi: 10.1371/journal.pone.0069146 (PMC3720533; doi:10.1371/journal.pone.0069146)
Supplement: Table S5 — Maximal fluxes rates, affinity, inhibition, threshold, stoichiometric ratios and other constants. (DOCX) [file pone.0069146.s005.docx]

**Table S5.**  Maximal fluxes (*V_m_*), affinity (*K_m_*), inhibition (*K_i_*), threshold (*TRH*), stoichiometric ratios (n) and other constants. Values were taken from BRENDA databank (http://www.brenda-enzymes.info/index.php4) for neurons or homo sapiens, or as specified otherwise. Enzyme E.C. (Enzyme Commission) numbers are given in brackets.

| Enzyme or reaction | Parameter | Value | Reference | Units |
| --- | --- | --- | --- | --- |
| Flux maximum rate |  |  |  |  |
| *Adenylate kinase* – forward reaction | $\boldsymbol{V}\boldsymbol{m}_{\boldsymbol{a}\boldsymbol{k}_{\boldsymbol{f}}}$ | 1300 | From model simulation | μM/min |
| *Adenylate kinase* – reverse reaction | $\boldsymbol{V}\boldsymbol{m}_{\boldsymbol{a}\boldsymbol{k}_{\boldsymbol{r}}}$ | 1000 | “ | μM/min |
| ATP consumption reactions | $\boldsymbol{V}\boldsymbol{m}_{\boldsymbol{atpase}}$ | 23500 | “ | μM/min |
| *Citrate dehydrogenase* | $\boldsymbol{V}\boldsymbol{m}_{\boldsymbol{cdh}}$ | 1824 | “ | μM/min |
| *Creatine kinase* – forward reaction | $\boldsymbol{V}\boldsymbol{m}_{\boldsymbol{c}\boldsymbol{k}_{\boldsymbol{f}}}$ | 6000 | “  “ | μM/min |
| *Creatine kinase* – reverse reaction | $\boldsymbol{V}\boldsymbol{m}_{\boldsymbol{c}\boldsymbol{k}_{\boldsymbol{r}}}$ | 450 | “ | μM/min |
| *Citrate synthase* | $\boldsymbol{V}\boldsymbol{m}_{\boldsymbol{cs}}$ | 2636 | “ | μM/min |
| Energetic metabolism stress recovery | $\boldsymbol{V}\boldsymbol{m}_{\boldsymbol{destress}}$ | 0.5 | “ | μM/min |
| *Fructose-6-phosphate aldolase* and *isomerase* – forward reaction | $\boldsymbol{V}\boldsymbol{m}_{\boldsymbol{fa}\boldsymbol{i}_{\boldsymbol{f}}}$ | 900 | “ | μM/min |
| *Fructose-6-phosphate aldolase* and *isomerase* – reverse reaction | $\boldsymbol{V}\boldsymbol{m}_{\boldsymbol{fa}\boldsymbol{i}_{\boldsymbol{r}}}$ | 100 | “ | μM/min |
| *Fructose bi-phosphatase* | $\boldsymbol{V}\boldsymbol{m}_{\boldsymbol{fbp}}$ | 0 | “ | μM/min |
| *Fumarate hydrogenase* | $\boldsymbol{V}\boldsymbol{m}_{\boldsymbol{fh}}$ | 2404 | “ | μM/min |
| *Glucose-6-phosphate dehydrogenase* | $\boldsymbol{V}\boldsymbol{m}_{\boldsymbol{g}\boldsymbol{6}\boldsymbol{d}}$ | 300 | “ | μM/min |
| *Glutamine synthase* – forward reaction | $\boldsymbol{V}\boldsymbol{m}_{\boldsymbol{gn}\boldsymbol{s}_{\boldsymbol{f}}}$ | 10 | “ | μM/min |
| *Glutamine synthase* – reverse reaction | $\boldsymbol{V}\boldsymbol{m}_{\boldsymbol{gn}\boldsymbol{s}_{\boldsymbol{r}}}$ | 20 | “ | μM/min |
| *Glutamate dehydronenase* – forward reaction | $\boldsymbol{V}\boldsymbol{m}_{\boldsymbol{gt}\boldsymbol{d}_{\boldsymbol{f}}}$ | 20 | “ | μM/min |
| *Glutamate dehydronenase* – reverse reaction | $\boldsymbol{V}\boldsymbol{m}_{\boldsymbol{gt}\boldsymbol{d}_{\boldsymbol{r}}}$ | 41 | “ | μM/min |
| *Glycogene phosphorylase* | $\boldsymbol{V}\boldsymbol{m}_{\boldsymbol{gyp}}$ | 2.337 | [32] | μM/min |
| *Glycogene synthase* | $\boldsymbol{V}\boldsymbol{m}_{\boldsymbol{gys}}$ | 3.27 | [32] | μM/min |
| *Glucose hexokinase* | $\boldsymbol{V}\boldsymbol{m}_{\boldsymbol{hk}}$ | 3000 | [32] | μM/min |
| *Glucose-6-phosphate isomerase* – forward reaction | $\boldsymbol{V}\boldsymbol{m}_{\boldsymbol{is}\boldsymbol{o}_{\boldsymbol{f}}}$ | 2500 | From model simulation | μM/min |
| *Glucose-6-phosphate isomerase* – reverse reaction | $\boldsymbol{V}\boldsymbol{m}_{\boldsymbol{is}\boldsymbol{o}_{\boldsymbol{r}}}$ | 1000 | “ | μM/min |
| *α-ketoglutarate dehydrogenase* | $\boldsymbol{V}\boldsymbol{m}_{\boldsymbol{kdh}}$ | 7549 | “ | μM/min |
| *Lactate dehydrogenase* - forward reaction | $\boldsymbol{V}\boldsymbol{m}_{\boldsymbol{ld}\boldsymbol{h}_{\boldsymbol{f}}}$ | 2000 | “ | μM/min |
| *Lactate dehydrogenase* - reverse reaction | $\boldsymbol{V}\boldsymbol{m}_{\boldsymbol{ld}\boldsymbol{h}_{\boldsymbol{r}}}$ | 5000 | “ | μM/min |
| Mitochondrial proton leak | $\boldsymbol{V}\boldsymbol{m}_{\boldsymbol{leak}}$ | 1200 | “ | μM/min |
| *Malate dehydrogenase* | $\boldsymbol{V}\boldsymbol{m}_{\boldsymbol{mdh}}$ | 2890 | “ | μM/min |
| *Oxydative phosphorylation* | $\boldsymbol{V}\boldsymbol{m}_{\boldsymbol{op}}$ | 20000 | “ | μM/min |
| *Pyruvate carboxylase* | $\boldsymbol{V}\boldsymbol{m}_{\boldsymbol{pc}}$ | 28 | “ | μM/min |
| *Pyruvate dehydrogenase* | $\boldsymbol{V}\boldsymbol{m}_{\boldsymbol{pdh}}$ | 2700 | “ | μM/min |
| *Phosphofructokinase* | $\boldsymbol{V}\boldsymbol{m}_{\boldsymbol{pfk}}$ | 3000 | “ | μM/min |
| *Phosphoglyceraldehyde kinase* | $\boldsymbol{V}\boldsymbol{m}_{\boldsymbol{pgk}}$ | 3000 | “ | μM/min |
| *Pyruvate kinase* | $\boldsymbol{V}\boldsymbol{m}_{\boldsymbol{pk}}$ | 10000 | [32] | μM/min |
| Pentose phosphate pathway | $\boldsymbol{V}\boldsymbol{m}_{\boldsymbol{ppp}}$ | 250 | From model simulation | μM/min |
| *Succinate dehydrogenase* | $\boldsymbol{V}\boldsymbol{m}_{\boldsymbol{sdh}}$ | 2000 | “ | μM/min |
| Energetic metabolism stress | $\boldsymbol{V}\boldsymbol{m}_{\boldsymbol{stress}}$ | 100 | “ | μM/min |
| Transmembrane transport of glucose | $\boldsymbol{V}\boldsymbol{m}_{\boldsymbol{t}_{\boldsymbol{glc}}}$ | 1050 | “ | μM/min |
| Transmembrane transport of glutamine | $\boldsymbol{V}\boldsymbol{m}_{\boldsymbol{t}_{\boldsymbol{gln}}}$ | 40 | “ | μM/min |
| Transmembrane transport of glutamate | $\boldsymbol{V}\boldsymbol{m}_{\boldsymbol{t}_{\boldsymbol{glt}}}$ | 22 | “ | μM/min |
| Transmembrane transport of lactate | $\boldsymbol{V}\boldsymbol{m}_{\boldsymbol{t}_{\boldsymbol{lac}}}$ | 120 | “ | μM/min |
|  |  |  |  |  |
|  |  |  |  |  |
|  |  |  |  |  |
|  |  |  |  |  |
| Table S5. (Continued) |  |  |  |  |
| Enzyme or reaction | **Parameter** | **Value** | **Reference** | **Units** |
| Affinity constant |  |  |  |  |
| *Adenylate kinase* for ADP | $\boldsymbol{K}\boldsymbol{m}_{\boldsymbol{a}\boldsymbol{k}_{\boldsymbol{adp}}}$ | 153 | From model simulation | μM |
| *Adenylate kinase* for AMP | $\boldsymbol{K}\boldsymbol{m}_{\boldsymbol{a}\boldsymbol{k}_{\boldsymbol{amp}}}$ | 276 | “ | μM |
| *Adenylate kinase* for ATP | $\boldsymbol{K}\boldsymbol{m}_{\boldsymbol{a}\boldsymbol{k}_{\boldsymbol{atp}}}$ | 148 | From model simulation | μM |
| *ATPase* for ATP | $\boldsymbol{K}\boldsymbol{m}_{\boldsymbol{atpas}\boldsymbol{e}_{\boldsymbol{atp}}}$ | 500 | [32] | μM |
| *Citrate dehydrogenase* for citrate | $\boldsymbol{K}\boldsymbol{m}_{\boldsymbol{cd}\boldsymbol{h}_{\boldsymbol{cit}}}$ | 275 | BRENDA [4.2.1.3] | μM |
| *Citrate dehydrogenase* for NAD | $\boldsymbol{K}\boldsymbol{m}_{\boldsymbol{cd}\boldsymbol{h}_{\boldsymbol{nad}}}$ | 74.33333 | BRENDA [4.2.1.3] | μM |
| *Creatine kinase* for ADP | $\boldsymbol{K}\boldsymbol{m}_{\boldsymbol{c}\boldsymbol{k}_{\boldsymbol{adp}}}$ | 40 | BRENDA [2.7.3.2] | μM |
| *Creatine kinase* for ATP | $\boldsymbol{K}\boldsymbol{m}_{\boldsymbol{c}\boldsymbol{k}_{a\boldsymbol{tp}}}$ | 635 | BRENDA [2.7.3.2] | μM |
| *Creatine kinase* for creatine | $\boldsymbol{K}\boldsymbol{m}_{\boldsymbol{c}\boldsymbol{k}_{\boldsymbol{cr}}}$ | 7500 | BRENDA [2.7.3.2] | μM |
| *Creatine kinase* for P-creatine | $\boldsymbol{K}\boldsymbol{m}_{\boldsymbol{c}\boldsymbol{k}_{\boldsymbol{pcr}}}$ | 510 | BRENDA [2.7.3.2] | μM |
| *Citrate synthase* for acetyl CoA | $\boldsymbol{K}\boldsymbol{m}_{\boldsymbol{c}\boldsymbol{s}_{\boldsymbol{aca}}}$ | 220 | BRENDA [2.3.3.1] | μM |
| *Citrate synthase* for oxaloacetate | $\boldsymbol{K}\boldsymbol{m}_{\boldsymbol{c}\boldsymbol{s}_{\boldsymbol{oaa}}}$ | 5.9 | BRENDA [2.3.3.1] | μM |
| Energetic metabolism affinity for adenosine-Ps under stress | $\boldsymbol{K}\boldsymbol{m}_{\boldsymbol{destres}\boldsymbol{s}_{\boldsymbol{anps}}}$ | 300 | From model simulation | μM |
| *Fructose-6-phosphate aldolase* and *isomerase* for fructose-biphosphate (fbp) | $\boldsymbol{K}\boldsymbol{m}_{\boldsymbol{fa}\boldsymbol{i}_{\boldsymbol{fbp}}}$ | 13.25 | BRENDA [4.1.2.13] | μM |
| *Fructose-6-phosphate aldolase* and *isomerase* for glyceraldehyde-3-phosphate | $\mathbf{K}\mathbf{m}_{\mathbf{fa}\mathbf{i}_{\mathbf{g3p}}}$ | 383.33 | BRENDA [5.3.1.1] | μM |
| *Fructose bi-phosphatase* for fbp | $\boldsymbol{K}\boldsymbol{m}_{\boldsymbol{fb}\boldsymbol{p}_{\boldsymbol{fbp}}}$ | 1.67 | BRENDA [3.1.3.11] | μM |
| *Fumarate hydrogenase* for fumarate | $\mathbf{K}\mathbf{m}_{\mathbf{f}\mathbf{h}_{f\mathbf{um}}}$ | 13 | BRENDA [4.2.1.2] | μM |
| *Glucose-6-phosphate dehydrogenase* for glucose-6-phosphate | $\boldsymbol{K}\boldsymbol{m}_{\boldsymbol{g}\boldsymbol{6}\boldsymbol{d}_{\boldsymbol{g}\boldsymbol{6}\boldsymbol{p}}}$ | 168.8 | BRENDA [1.1.1.49] | μM |
| *Glucose-6-phosphate dehydrogenase* for NADP | $\boldsymbol{K}\boldsymbol{m}_{\boldsymbol{g}\boldsymbol{6}\boldsymbol{d}_{\boldsymbol{nadp}}}$ | 10.035 | BRENDA [1.1.1.49] | μM |
| *Glutamine synthase* for ADP | $\boldsymbol{K}\boldsymbol{m}_{\boldsymbol{gn}\boldsymbol{s}_{\boldsymbol{adp}}}$ | 612.3125 | BRENDA [6.3.2.1] | μM |
| *Glutamine synthase* for ATP | $\boldsymbol{K}\boldsymbol{m}_{\boldsymbol{gn}\boldsymbol{s}_{\boldsymbol{atp}}}$ | 2000 | BRENDA [6.3.2.1] | μM |
| *Glutamine synthase* for glutamine | $\boldsymbol{K}\boldsymbol{m}_{\boldsymbol{gn}\boldsymbol{s}_{\boldsymbol{gln}}}$ | 22772.73 | BRENDA [6.3.2.1] | μM |
| *Glutamine synthase* for glutamate | $\boldsymbol{K}\boldsymbol{m}_{\boldsymbol{gn}\boldsymbol{s}_{\boldsymbol{glt}}}$ | 1200 | BRENDA [6.3.2.1] | μM |
| *Glutamate dehydronenase* for α-ketoglutarate | $\boldsymbol{K}\boldsymbol{m}_{\boldsymbol{gt}\boldsymbol{d}_{\boldsymbol{akg}}}$ | 1416.154 | BRENDA [1.4.1.3] | μM |
| *Glutamate dehydronenase* for glutamate | $\boldsymbol{K}\boldsymbol{m}_{\boldsymbol{gt}\boldsymbol{d}_{\boldsymbol{glt}}}$ | 5298.333 | BRENDA [1.4.1.3] | μM |
| *Glutamate dehydronenase* for NAD | $\boldsymbol{K}\boldsymbol{m}_{\boldsymbol{gt}\boldsymbol{d}_{\boldsymbol{nad}}}$ | 502.5 | BRENDA [1.4.1.3] | μM |
| *Glutamate dehydronenase* for NADH | $\boldsymbol{K}\boldsymbol{m}_{\boldsymbol{gt}\boldsymbol{d}_{\boldsymbol{nadh}}}$ | 75.36364 | BRENDA [1.4.1.3] | μM |
| *Glycogene phosphorylase* for glycogen | $\boldsymbol{K}\boldsymbol{m}_{\boldsymbol{gy}\boldsymbol{p}_{\boldsymbol{gly}}}$ | 1003.242 | [32] | μM |
| *Glycogene synthase* for ATP | $\boldsymbol{K}\boldsymbol{m}_{\boldsymbol{gy}\boldsymbol{s}_{\boldsymbol{atp}}}$ | 74 | BRENDA [5.4.2.2] | μM |
| *Glycogene synthase* for *glucose-6-phosphate* | $\boldsymbol{K}\boldsymbol{m}_{\boldsymbol{gy}\boldsymbol{s}_{\boldsymbol{g}\boldsymbol{6}\boldsymbol{p}}}$ | 500 | [32] | μM |
| *Glucose hexokinase* for ATP | $\boldsymbol{K}\boldsymbol{m}_{\boldsymbol{h}\boldsymbol{k}_{\boldsymbol{atp}}}$ | 592 | BRENDA [2.7.1.1] | μM |
| *Glucose hexokinase* for glucose | $\boldsymbol{K}\boldsymbol{m}_{\boldsymbol{h}\boldsymbol{k}_{\boldsymbol{glc}}}$ | 0.0775 | [32] | μM |
| *Glucose-6-phosphate isomerase* for *fructose-6-phosphate* | $\boldsymbol{K}\boldsymbol{m}_{\boldsymbol{is}\boldsymbol{o}_{\boldsymbol{f}\boldsymbol{6}\boldsymbol{p}}}$ | 0.06078 | [32] | μM |
| *Glucose-6-phosphate isomerase* for *glucose-6-phosphate* | $\boldsymbol{K}\boldsymbol{m}_{\boldsymbol{is}\boldsymbol{o}_{\boldsymbol{g}\boldsymbol{6}\boldsymbol{p}}}$ | 423.75 | [32] | μM |
| *α-ketoglutarate dehydrogenase* for ADP | $\boldsymbol{K}\boldsymbol{m}_{\boldsymbol{kd}\boldsymbol{h}_{\boldsymbol{adp}}}$ | 125.9 | BRENDA [1.2.4.2] | μM |
| *α-ketoglutarate dehydrogenase* for *α-ketoglutarate* | $\boldsymbol{K}\boldsymbol{m}_{\boldsymbol{kd}\boldsymbol{h}_{\boldsymbol{akg}}}$ | 13 | BRENDA [1.2.4.2] | μM |
| *α-ketoglutarate dehydrogenase* for NAD | $\boldsymbol{K}\boldsymbol{m}_{\boldsymbol{kd}\boldsymbol{h}_{\boldsymbol{nad}}}$ | 280 | BRENDA [1.8.1.4] | μM |
| *α-ketoglutarate dehydrogenase* for NADH | $\boldsymbol{K}\boldsymbol{m}_{\boldsymbol{kd}\boldsymbol{h}_{\boldsymbol{nadh}}}$ | 61 | BRENDA [1.8.1.4] | μM |
| *Lactate dehydrogenase* for lactate | $\boldsymbol{K}\boldsymbol{m}_{\boldsymbol{ld}\boldsymbol{h}_{\boldsymbol{lac}}}$ | 1790 | BRENDA [1.1.1.27] | μM |
| *Lactate dehydrogenase* for NAD | $\boldsymbol{K}\boldsymbol{m}_{\boldsymbol{ld}\boldsymbol{h}_{\boldsymbol{nad}}}$ | 1100 | BRENDA [1.1.1.27] | μM |
| *Lactate dehydrogenase* for NADH | $\boldsymbol{K}\boldsymbol{m}_{\boldsymbol{ld}\boldsymbol{h}_{\boldsymbol{nadh}}}$ | 6 | BRENDA [1.1.1.27] | μM |
| *Lactate dehydrogenase* for pyruvate | $\boldsymbol{K}\boldsymbol{m}_{\boldsymbol{ld}\boldsymbol{h}_{\boldsymbol{pyr}}}$ | 214 | BRENDA [1.1.1.27] | μM |
| Mitochondrial proton leak for NADH | $\boldsymbol{K}\boldsymbol{m}_{\boldsymbol{lea}\boldsymbol{k}_{\boldsymbol{nadh}}}$ | 200 | From model simulation | μM |
| *Malate dehydrogenase* for malate | $\boldsymbol{K}\boldsymbol{m}_{\boldsymbol{md}\boldsymbol{h}_{\boldsymbol{mal}}}$ | 500 | BRENDA [1.1.1.37] | μM |
| *Malate dehydrogenase* for NAD | $\boldsymbol{K}\boldsymbol{m}_{\boldsymbol{md}\boldsymbol{h}_{\boldsymbol{na}d}}$ | 140 | BRENDA [1.1.1.37] | μM |
| *Oxydative phosphorylation* for ADP | $\boldsymbol{K}\boldsymbol{m}_{\boldsymbol{o}\boldsymbol{p}_{\boldsymbol{adp}}}$ | 1.0735 | [32] | μM |
| Table S5. (Continued) |  |  |  |  |
| Enzyme or reaction | **Parameter** | **Value** | **Reference** | **Units** |
| Affinity constant |  |  |  |  |
| *Oxydative phosphorylation* for NADH | $\boldsymbol{K}\boldsymbol{m}_{\boldsymbol{o}\boldsymbol{p}_{\boldsymbol{nadh}}}$ | 2.75 | From model simulation | μM |
| *Oxydative phosphorylation* for O_2_ | $\boldsymbol{K}\boldsymbol{m}_{\boldsymbol{o}\boldsymbol{p}_{\boldsymbol{o}\boldsymbol{2}}}$ | 2.9658 | [32] | μM |
| *Pyruvate carboxylase* for ATP | $\boldsymbol{K}\boldsymbol{m}_{\boldsymbol{p}\boldsymbol{c}_{\boldsymbol{atp}}}$ | 235 | BRENDA [6.4.1.1] | μM |
| *Pyruvate carboxylase* for pyruvate | $\boldsymbol{K}\boldsymbol{m}_{\boldsymbol{p}\boldsymbol{c}_{\boldsymbol{pyr}}}$ | 162.5 | BRENDA [6.4.1.1] | μM |
| *Pyruvate dehydrogenase* for coenzyme A | $\boldsymbol{K}\boldsymbol{m}_{\boldsymbol{pd}\boldsymbol{h}_{\boldsymbol{coa}}}$ | 1 | From model simulation | μM |
| *Pyruvate dehydrogenase* for NAD | $\boldsymbol{K}\boldsymbol{m}_{\boldsymbol{pd}\boldsymbol{h}_{\boldsymbol{nad}}}$ | 280 | BRENDA [1.8.1.4] | μM |
| *Pyruvate dehydrogenase* for pyruvate | $\boldsymbol{K}\boldsymbol{m}_{\boldsymbol{pd}\boldsymbol{h}_{\boldsymbol{pyr}}}$ | 36.9 | BRENDA [1.2.4.1] | μM |
| *Phosphofructokinase* for ATP | $\boldsymbol{K}\boldsymbol{m}_{\boldsymbol{pf}\boldsymbol{k}_{\boldsymbol{atp}}}$ | 120 | BRENDA [2.7.1.11] | μM |
| *Phosphofructokinase* for *fructose-6-phosphate* | $\boldsymbol{K}\boldsymbol{m}_{\boldsymbol{pf}\boldsymbol{k}_{\boldsymbol{f}\boldsymbol{6}\boldsymbol{p}}}$ | 113.5 | [32] | μM |
| *Phosphoglyceraldehyde kinase* for ADP | $\boldsymbol{K}\boldsymbol{m}_{\boldsymbol{pg}\boldsymbol{k}_{\boldsymbol{adp}}}$ | 142 | BRENDA [2.7.2.3] | μM |
| *Phosphoglyceraldehyde kinase* for glyceraldehyde-3-phosphate | $\boldsymbol{K}\boldsymbol{m}_{\boldsymbol{pg}\boldsymbol{k}_{\boldsymbol{g}\boldsymbol{3}\boldsymbol{p}}}$ | 70 | BRENDA [2.7.2.3] | μM |
| *Pyruvate kinase* for ADP | $\boldsymbol{K}\boldsymbol{m}_{\boldsymbol{p}\boldsymbol{k}_{\boldsymbol{adp}}}$ | 340 | BRENDA [2.7.1.40] | μM |
| *Pyruvate kinase* for phosphoenolpyruvate | $\boldsymbol{K}\boldsymbol{m}_{\boldsymbol{p}\boldsymbol{k}_{\boldsymbol{pep}}}$ | 20 | From model simulation | μM |
| Pentose Phosphate Pathway for ribulose-5-phosphate | $Km_{ppp_{r5p}}$ | 250 | From model simulation | μM |
| *Succinate dehydrogenase* for NAD | $\boldsymbol{K}\boldsymbol{m}_{\boldsymbol{sd}\boldsymbol{h}_{\boldsymbol{nad}}}$ | 193.583 | BRENDA [1.3.5.1] | μM |
| *Succinate dehydrogenase* for succinate | $\boldsymbol{K}\boldsymbol{m}_{\boldsymbol{sd}\boldsymbol{h}_{\boldsymbol{suc}}}$ | 371.13 | BRENDA [1.3.5.1] | μM |
| Energetic metabolism stress for ATP | $\boldsymbol{K}\boldsymbol{m}_{\boldsymbol{stres}\boldsymbol{s}_{\boldsymbol{atp}}}$ | 300 | From model simulation | μM |
| Glucose transmembrane transporter | $\boldsymbol{K}\boldsymbol{m}_{\boldsymbol{t}_{\boldsymbol{glc}}}$ | 4340.485 | [32] | μM |
| Glutamine transmembrane transporter | $\boldsymbol{K}\boldsymbol{m}_{\boldsymbol{t}_{\boldsymbol{gln}}}$ | 434 | From model simulation | μM |
| Glutamate transmembrane transporter | $\boldsymbol{K}\boldsymbol{m}_{\boldsymbol{t}_{\boldsymbol{glt}}}$ | 12 | From model simulation | μM |
| Lactate transmembrane transporter | $\boldsymbol{K}\boldsymbol{m}_{\boldsymbol{t}_{\boldsymbol{la}c}}$ | 439.348 | [32] | μM |
| Inhibition constant of ATP on p*hosphofructokinase* | $\boldsymbol{K}\boldsymbol{i}_{\boldsymbol{pf}\boldsymbol{k}_{\boldsymbol{atp}}}$ | 759.49 | [32] | μM |
| Concentration threshold level |  |  |  |  |
| Inhibition of glycogen on the glycogen buffer pathway | $\boldsymbol{TR}\boldsymbol{H}_{\boldsymbol{i}_{\boldsymbol{gy}\boldsymbol{s}_{\boldsymbol{gly}}}}$ | 4200 | [32] | μM |
| Inhibition of G6P on *hexokinase* | $\boldsymbol{TR}\boldsymbol{H}_{\boldsymbol{i}_{\boldsymbol{h}\boldsymbol{k}_{\boldsymbol{g}\boldsymbol{6}\boldsymbol{p}}}}$ | 600 | [32] | μM |
| Inhibition of a stress on ATP production | $\boldsymbol{TR}\boldsymbol{H}_{\boldsymbol{i}_{\boldsymbol{stres}\boldsymbol{s}_{\boldsymbol{atp}}}}$ | 300 | From model simulation | μM |
| Other constants, experimental data and stoichiometric ratios |  |  |  |  |
| Oxygen concentration in extracellular medium | $\boldsymbol{O2}\boldsymbol{e}_{\boldsymbol{0}}$ | 1235 | Experimental | μM |
| Initial brain slices volume in a Petri plate | $\boldsymbol{Cell}\boldsymbol{s}_{\boldsymbol{0}}$ | 0.000548 | Experimental data | L |
| Brain slices volume in a sample | $\boldsymbol{Cell}\boldsymbol{s}_{\mathbf{m}}$ | 0.000069 | Experimental data | L |
| Oxidative phosphorylation capacity (0.90 for Parkin KO mice) | $\boldsymbol{Et}\boldsymbol{a}_{\boldsymbol{op}}$ | 0.99 | From model simulation | - |
|  |  |  |  |  |
| Stoichiometric coefficient from FAI to G3P | $\boldsymbol{n}_{\boldsymbol{fa}\boldsymbol{i}_{\boldsymbol{g}\boldsymbol{3}\boldsymbol{p}}}$ | 2 | [34] | - |
| Stoichiometric coefficient form G6PD and 6PGnD | $\boldsymbol{n}_{\boldsymbol{g}\boldsymbol{6}\boldsymbol{d}_{\boldsymbol{nadph}}}$ | 2 | [34] | - |
| Stoichiometric coefficient of H^+^ leak: 1 NADH per O_2_ consumed | $\boldsymbol{n}_{\boldsymbol{lea}\boldsymbol{k}_{\boldsymbol{o}\boldsymbol{2}}}$ | 0.5 | [34] | - |
| Stoichiometric coefficient of respiration: ATP per NADH | $\boldsymbol{n}_{\boldsymbol{o}\boldsymbol{p}_{\boldsymbol{atp}}}$ | 3.33 | From model simulation | - |
| Stoichiometric coefficient of respiration: O_2_ per NADH | $\boldsymbol{n}_{\boldsymbol{o}\boldsymbol{p}_{\boldsymbol{o}\boldsymbol{2}}}$ | 0.5 | [34] | - |
| Stoichiometric flux ratio from F6P to PPP | $\boldsymbol{n}_{\boldsymbol{pp}\boldsymbol{p}_{\boldsymbol{f}\boldsymbol{6p}}}$ | 0.666667 | From model simulation | - |
| Stoichiometric flux ratio from PPP to G3P | $\boldsymbol{n}_{\boldsymbol{pp}\boldsymbol{p}_{\boldsymbol{g}\boldsymbol{3}\boldsymbol{p}}}$ | 0.333333 | From model simulation | - |
| Stoichiometric flux ratio: NADH from *succinate dehydrogenase* | $\boldsymbol{n}_{\boldsymbol{sd}\boldsymbol{h}_{\boldsymbol{nadh}}}$ | 0.666667 | From model simulation | - |
| Stoichiometric flux ratio: NADH on p*hosphofructokinase* | $\boldsymbol{n}\boldsymbol{H}_{\boldsymbol{pfk}}$ | 4 | [32] | - |
|  |  |  |  |  |
| Volumetric rate of evaporation with time | $\boldsymbol{K}_{\boldsymbol{evap}}$ | 0.000032 | Experimental data | L/min |
| Volumetric rate of sampling with time | $\boldsymbol{K}_{\boldsymbol{sampling}}$ | 0.000007 | Experimental data | L/min |
| Specific mass transfer coefficient of O_2_ in the brain slice | $\boldsymbol{K}_{\boldsymbol{t}_{\boldsymbol{o}\boldsymbol{2}}}$ | 2644 | From model simulation | 1/min |
