# Supplementary material for: Metabolomics and In-Silico Analysis Reveal Critical Energy Deregulations in Animal Models of Parkinson’s Disease
Source: PLoS One. 2013 Jul 23;8(7):e69146. doi: 10.1371/journal.pone.0069146 (PMC3720533; doi:10.1371/journal.pone.0069146)
Supplement: Table S6 — Model Matlab® code. (DOCX) [file pone.0069146.s006.docx]

**Table S6.** Model Matlab® code

********** MODEL NAME

Brain Slices Energy Metabolism

********** MODEL NOTES

+++ OVERVIEW

This model describes the dynamics of energy metabolism in mice brain slices

under various stresses, including Parkinson's related toxins.

The model does not discriminate cell types in the brain and is thus

representative of overall brain tissue metabolism.

This implementation reproduces the result of brain slice exposure to

toxins, as presented in Figure 2 of the associated manuscript (see below)

The model is presented in :

Poliquin PO, Cloutier M, Perrier M, Trudeau LE, and Jolicoeur M,

Metabolomics and Mathematical Modelling Reveal Critical Energy

Deregulations in Animal Models of Parkinson's Disease.

PLoS ONE (2013)

Please cite the original article if you use this model

+++ POSSILE SIMULATIONS

- Mitochondria stress (this file) : CCCP Ionophore

- Transport stress : Hypoxia

- Oxydative stress : ROS

- Activity stress : ATPases

+++ STATES

- Transport : 7 (GLCe, LACe, GLTe, GLNe, O2e, V, CellsØ)

- Glycolysis : 7 (GLC, G6P, F6P, FBP, G3P, PEP, PYR)

- Glycogen buffer : 1 (GLY)

- Pentose phosphates : 1 (R5P)

- Oxydative stress response : 2 (NADPH, NADPØ)

- Stress response : 1 (ANPs)

- Lactate shuttle : 1 (LAC)

- TCA cycle : 8 (ACA, CIT, AKG, SUC, FUM, MAL, OAA, CoAØ)

- Glutamine cycling : 2 (GLN, GLT)

- Creatine buffer : 2 (PCr, CrØ)

- Energy : 6 (AMP, ADPØ, ATP, O2, NADH, NADØ)

- - Total of : 38

+++ FLUX

- Transport : 11 (T_glc, T_lac, T_glt, T_gln, T_o2, C_glc, C_lac, C_glt, C_gln, V_evap, T_o2e)

- Glycolysis : 6 (V_hk, V_iso, V_pfk, V_fai, V_pgk, V_pk)

- Futile cycling : 2 (V_fbp, V_leak)

- Glycogen buffer : 2 (V_gys, V_gyp)

- Pentose phosphates : 2 (V_g6d, V_ppp)

- Oxydative stress response : 1 (V_os)

- Stress response : 2 (V_stress, V_destress)

- TCA cycle : 8 (V_pdh, V_pc, V_cs, V_cdh, V_kdh, V_sdh, V_fh, V_mdh)

- Creatine buffer : 1 (V_ck)

- Lactate shuttle : 1 (V_ldh)

- Glutamine cycling : 2 (V_gtd, V_gns)

- Energy : 3 (V_ak, V_atpase, V_op)

- - Total of : 41

+++ PARAMETERS

- Experimentally fitted : 46

- Found experimentally : 5

- - Total of : 51

+++ CONSTANTS

- From litterature : 82

- For simulation : 28

- - Total of : 110

+++ COMPARTMENTS

- Intracellular : (Cytosol and mitochondria)

- Extracellular : (Medium)

+++ UNITS

- Time : minutes (min)

- Concentration : micromolar (uM)

- Volume : liters (L)

+++ CONTACT INFORMATION

Implemented in the Systems Biology Toolbox (v2) for Matlab

by Pierre-Olivier.Poliquin@polymtl.ca (2009-2012), supervised by

Mario Jolicoeur and Mathieu Cloutier

Curation and maintenance by Mathieu Cloutier

Please forward any inquiry to mathieu.cloutier@polymtl.ca or

mario.jolicoeur@polymtl.ca

********** MODEL STATES

d/dt(ACA) = +V_pdh-V_cs

d/dt(AKG) = +V_cdh-V_kdh+V_gtd

d/dt(AMP) = -V_ak+V_destress

d/dt(ANPs) = +V_stress-V_destress

d/dt(ATP) = -V_hk-V_pfk+V_pgk+V_pk-V_pc+V_kdh-V_gns-V_gys-V_ck+Eta_op*n_op_atp*V_op-V_atpase-V_ak-V_stress

d/dt(CIT) = +V_cs-V_cdh

d/dt(F6P) = +V_iso-V_pfk+n_ppp_f6p*V_ppp+V_fbp

d/dt(FBP) = +V_pfk-V_fai-V_fbp

d/dt(FUM) = +V_sdh-V_fh

d/dt(G3P) = +n_fai_g3p*V_fai-V_pgk+V_ppp

d/dt(G6P) = +V_hk-V_iso-n_ppp_g3p*V_g6d-V_gys+V_gyp

d/dt(GLC) = +T_glc-V_hk

d/dt(GLCe) = -R_volume*T_glc+C_glc

d/dt(GLN) = -T_gln+V_gns

d/dt(GLNe) = +R_volume*T_gln+C_gln

d/dt(GLT) = -T_glt-V_gtd-V_gns

d/dt(GLTe) = +R_volume*T_glt+C_glt

d/dt(GLY) = +V_gys-V_gyp

d/dt(LAC) = -T_lac-V_ldh

d/dt(LACe) = +R_volume*T_lac+C_lac

d/dt(MAL) = +V_fh-V_mdh

d/dt(NADH) = +V_pgk+V_ldh+V_pdh+V_cdh+V_kdh+n_sdh_nadh*V_sdh+V_mdh+V_gtd-V_leak-V_op

d/dt(NADPH) = +n_g6d_nadph*V_g6d-V_os

d/dt(O2) = +T_o2-n_op_o2*V_op-n_leak_o2*V_leak

d/dt(O2e) = +T_o2e

d/dt(OAA) = +V_mdh-V_cs+V_pc

d/dt(PCr) = +V_ck

d/dt(PEP) = +V_pgk-V_pk

d/dt(PYR) = +V_pk-V_pdh-V_pc+V_ldh

d/dt(R5P) = +V_g6d-V_ppp

d/dt(SUC) = +V_kdh-V_sdh

d/dt(V) = -V_evap

ACA(0) = 74.5

AKG(0) = 596

AMP(0) = 254

ANPs(0) = 0

ATP(0) = 872

CIT(0) = 290

F6P(0) = 130

FBP(0) = 18.65

FUM(0) = 11.36

G3P(0) = 14.89

G6P(0) = 644.95

GLC(0) = 1000

GLCe(0) = 9237

GLN(0) = 20

GLNe(0) = 4

GLT(0) = 200

GLTe(0) = 18

GLY(0) = 2635.5

LAC(0) = 400

LACe(0) = 120

MAL(0) = 596

NADH(0) = 230

NADPH(0) = 65.08

O2(0) = 1234.1

O2e(0) = 1235

OAA(0) = 5.39

PCr(0) = 1826.3

PEP(0) = 6.3

PYR(0) = 49

R5P(0) = 21.5

SUC(0) = 500

V(0) = 0.04

********** MODEL PARAMETERS

A_p_atpase = 0 %ATP pulse amplitude% Process: Energy

A_p_leak = 0 %Pulse amplitude - CCCP stress --> model hypothesis% Process: Energy

A_p_o2 = 0 %O2 Transfert pulse amplitude - O2 depletion --> model hypothesis% Process: Transport

A_p_os = 0 %Pulse amplitude - Oxydative stress% Process: Stress response

Cells_0 = 0.000548 %From lab% Process: Transport

Cells_min = 6.9e-005 %From lab% Process: Transport

Eta_op = 0.99 %Efficiency of respiratory transport chain for oxygen consumption (versus H2O2)% Process: Energy

F_i_gys_gly = 0.02 % Process: Glycogen buffer

F_i_hk_g6p = 0.02 % Process: Glycolysis

F_i_stress_atp = 0.02 % Process: Energy

F_p_off_atpase = 1 %Pulse% Process: Energy

F_p_off_leak = 1 %Pulse% Process: Energy

F_p_off_o2 = 1 %O2 transfert pulse force off% Process: Transport

F_p_off_os = 1 %Pulse% Process: Stress response

F_p_on_atpase = 1 %Pulse% Process: Energy

F_p_on_leak = 1 %Pulse% Process: Energy

F_p_on_o2 = 1 %O2 transfert pulse force on% Process: Transport

F_p_on_os = 1 %Pulse% Process: Stress response

K_evap = 3.2e-005 % Process: Transport

K_L_o2 = 500 %fitted% Process: Transport

K_os = 0.08 %hypothesis (should be same as PPPox)% Process: Stress response

K_sampling = 7e-006 %From lab% Process: Transport

K_t_o2 = 2644 % Process: Transport

Ki_pfk_atp = 759.49 % Process: Glycolysis

Km_ak_adp = 153 % Process: Energy

Km_ak_amp = 276 % Process: Energy

Km_ak_atp = 148 % Process: Energy

Km_atpase_atp = 500 % Process: Energy

Km_cdh_cit = 275 % Process: TCA

Km_cdh_nad = 74.33 % Process: TCA

Km_ck_adp = 40 %Process: Creatine buffer

Km_ck_atp = 635 %Process: Creatine buffer

Km_ck_cr = 7500 %Process: Creatine buffer

Km_ck_pcr = 510 %Process: Creatine buffer

Km_cs_aca = 220 %Process: TCA

Km_cs_oaa = 7.779 %Process: TCA

Km_destress_anps = 300 %Process: Energy

Km_fai_fbp = 13.25 %Process: Glycolysis

Km_fai_g3p = 383.33 %Process: Glycolysis

Km_fbp_fbp = 1.67 %Process: Futile cycling

Km_fh_fum = 13 %Process: TCA

Km_g6d_g6p = 168.8 %Process: Pentose phosphates

Km_g6d_nadp = 10.035 %Process: Pentose phosphates

Km_gns_adp = 612.3125 %Process: Glutam*

Km_gns_atp = 2000 %Process: Glutam*

Km_gns_gln = 22772.727273 %Process: Glutam*

Km_gns_glt = 1200 %Process: Glutam*

Km_gtd_akg = 1416.153846 %Process: Glutam*

Km_gtd_glt = 5298.33333 %Process: Glutam*

Km_gtd_nad = 502.5 %Process: Glutam*

Km_gtd_nadh = 75.363636 %Process: Glutam*

Km_gyp_gly = 1003.241667 %Process: Glycolysis

Km_gys_atp = 74 %Process: Glycogen buffer

Km_gys_g6p = 500 %Process: Glycogen buffer

Km_hk_atp = 592 %Process: Glycolysis

Km_hk_glc = 0.0775 % Process: Glycolysis

Km_iso_f6p = 0.06078 %Process: Glycolysis

Km_iso_g6p = 423.75 %Process: Glycolysis

Km_kdh_adp = 125.9 %Process: TCA

Km_kdh_akg = 13 %Process: TCA

Km_kdh_nad = 280 %Process: TCA

Km_kdh_nadh = 61 %Process: TCA

Km_ldh_lac = 1790 %Process: Lactate

Km_ldh_nad = 1100 %Process: Lactate

Km_ldh_nadh = 60 %Process: Lactate

Km_ldh_pyr = 214 %Process: Lactate

Km_leak_nadh = 200 %hypothesis% Process: Energy

Km_mdh_mal = 500 %Process: TCA

Km_mdh_nad = 140 %Process: TCA

Km_op_adp = 1.0735 %Process: Energy

Km_op_nadh = 2.75 %Affinity constant of NADH with OP (nMol) hypothesis% Process: Energy

Km_op_o2 = 2.9658 %Process: Energy

Km_pc_atp = 235 %Process: TCA

Km_pc_pyr = 162.5 %Process: TCA

Km_pdh_coa = 1 %hypothesis% Process: TCA

Km_pdh_nad = 280 %Process: TCA

Km_pdh_pyr = 36.9 %Process: TCA

Km_pfk_atp = 120 %Process: Glycolysis

Km_pfk_f6p = 113.5 %Process: Glycolysis

Km_pgk_adp = 142 %Process: Glycolysis

Km_pgk_g3p = 70 %Process: Glycolysis

Km_pk_adp = 340 %Process: Glycolysis

Km_pk_pep = 20 %Fitted% Process: Glycolysis

Km_ppp_r5p = 250 %Hypothesis% Process: Pentose phosphates

Km_sdh_nad = 193.583 %Process: TCA

Km_sdh_suc = 371.13 %Process: TCA

Km_stress_atp = 300 %Process: Energy

Km_t_glc = 4340.485 %Process: Transport

Km_t_gln = 434 %Process: Transport

Km_t_glt = 12 %Process: Transport

Km_t_lac = 439.348 %Process: Transport

Km_t_o2 = 0.351513 %(in atm x 760mmHg, x1.3E-3 mM/mmHg in water @ RT x1000mM x1000uM% Process: Transport

n_fai_g3p = 2 %Stoechiometric ratio for G3P formation by FAI as (DHAP is converted at the same% Process: Glycolysis

n_g6d_nadph = 2 %Stoechiometric ratio for NADPH formation by PPP (G6PD and 6PGnD)% Process: Pentose phosphates

n_leak_o2 = 0.5 %Stoechiometric ratio for O2 consumption by LEAK (based on 1 NADH)% Process: Energy

n_op_atp = 3.33 %Stoechiometric ratio for ATP formation by OP (based on 1 NADH)% Process: Energy

n_op_o2 = 0.5 %Stoechiometric ratio for O2 consumption by OP (based on 1 NADH)% Process: Energy

n_ppp_f6p = 0.666667 %Flux ratio for mass balance% Process: Pentose phosphates

n_ppp_g3p = 0.333333 %Flux ratio for mass balance% Process: Pentose phosphates

n_sdh_nadh = 0.666667 %Stoechiometric ratio for NADH generation (2/3 for SDH)% Process: TCA

nH_pfk = 4 %Process: Glycolysis

O2e_0 = 1235 %From lab% Process: Energy

P_p_off_atpase = 0.01 %Hypothesis% Process: Energy

P_p_off_leak = 0.01 %Hypothesis% Process: Energy

P_p_off_o2 = 0.01 %Hypothesis% Process: Transport

P_p_off_os = 0.01 %Hypothesis% Process: Pentose phosphates

P_p_on_atpase = 0.01 %Hypothesis% Process: Energy

P_p_on_leak = 0.01 %Hypothesis% Process: Energy

P_p_on_o2 = 0.01 %Hypothesis% Process: Transport

P_p_on_os = 0.01 %Hypothesis% Process: Pentose phosphates

T_p_off_atpase = 35 %Pulse% Process: Energy

T_p_off_leak = 35 %Pulse% Process: Energy

T_p_off_o2 = 35 %O2 transfert pulse turn off time% Process: Transport

T_p_off_os = 35 %Pulse% Process: Stress response

T_p_on_atpase = 15 %Pulse% Process: Energy

T_p_on_leak = 15 %Pulse% Process: Energy

T_p_on_o2 = 15 %O2 transfert pulse turn on time% Process: Transport

T_p_on_os = 15 %Pulse% Process: Stress response

TRH_i_gys_gly = 4200 %Process: Glycogen buffer

TRH_i_hk_g6p = 600 %Process: Glycolysis

TRH_i_stress_atp = 300 %Hypothesis% Process: Energy

Vm_ak_f = 1300 %Fitted% Process: Energy

Vm_ak_r = 1000 %Fitted% Process: Energy

Vm_atpase = 23500 %Fitted% Process: Energy

Vm_cdh = 2636.4821418405695 %Fitted% Process: TCA

Vm_ck_f = 6000 %Fitted% Process: Creatine buffer

Vm_ck_r = 450 %Fitted% Process: Creatine buffer

Vm_cs = 4177.778455327496 %Fitted% Process: TCA

Vm_destress = 0.5 %hypothesis% Process: Energy

Vm_fai_f = 900 %Fitted% Process: Glycolysis

Vm_fai_r = 100 %Fitted% Process: Glycolysis

Vm_fbp = 0 %Hypothesis% Process: Futile cycling

Vm_fh = 957.049638 %Fitted% Process: TCA

Vm_g6d = 300 %Fitted% Process: Pentose phosphates

Vm_gns_f = 10 %Hypothesis% Process: Glutam*

Vm_gns_r = 20 %Hypothesis% Process: Glutam*

Vm_gtd_f = 20 %Hypothesis% Process: Glutam*

Vm_gtd_r = 41 %Hypothesis% Process: Glutam*

Vm_gyp = 2.337 %Process: Glycogen buffer

Vm_gys = 3.27 %Process: Glycogen buffer

Vm_hk = 3000 %Process: Glycolysis

Vm_iso_f = 2500 %Fitted% Process: Glycolysis

Vm_iso_r = 1000 %Fitted% Process: Glycolysis

Vm_kdh = 7549 %Fitted% Process: TCA

Vm_ldh_f = 5023.772863019155 %Fitted% Process: Lactate

Vm_ldh_r = 659.12836927820229 %Fitted% Process: Lactate

Vm_leak = 1200 %hypothesis% Process: Energy

Vm_mdh = 2890 %Fitted% Process: TCA

Vm_op = 20000 %Fitted% Process: Energy

Vm_pc = 28 %Fitted% Process: TCA

Vm_pdh = 4279.21 %Hypothesis% Process: TCA

Vm_pfk = 3000 %Fitted% Process: Glycolysis

Vm_pgk = 3000 %Fitted% Process: Glycolysis

Vm_pk = 10000 % Process: Glycolysis

Vm_ppp = 250 %Hypothesis% Process: Pentose phosphates

Vm_sdh = 6039.90 %Fitted% Process: TCA

Vm_stress = 100 %hypothesis% Process: Stress response

Vm_t_glc = 1050 %Fitted% Process: Transport

Vm_t_gln = 40 %Fitted% Process: Transport

Vm_t_glt = 22 %Fitted% Process: Transport

Vm_t_lac = 1094.41300722709 % Process: Transport

ANP_T = 1300.76 %uM % Energy %

CoA_T = 76.3 %uM % TCA % total Coenzyme A

Cr_T = 2500 %uM % Creatine buffer

NAD_T = 1070 %uM % Energy

NADP_T = 66 %uM % Energy %

********** MODEL VARIABLES

ADP = ANP_T-ATP-AMP-ANPs %uM % Energy %

ANP = ANP_T-ANPs %uM % %

Cells = max(Cells_0-K_sampling*time,Cells_min) %L % Transport % from lab (average)

CoA = CoA_T-ACA %uM % TCA %

Cr = Cr_T-PCr %uM % Creatine buffer %

NAD = NAD_T-NADH %uM % Energy %

NADP = NADP_T-NADPH %uM % Energy %

ATP_ANP_T = ATP/ANP_T %% % Ratio %

NADH_NAD = NADH/NAD %% % Ratio %

NADH_NAD_T = NADH/NAD_T %% % Ratio %

NADPH_NADP = NADPH/NADP %% % Ratio %

NADPH_NADP_T = NADPH/NADP_T %% % Ratio %

PCR_Cr_T = PCr/Cr_T %% % Ratio %

R_volume = Cells/V %% % Ratio %

C_glc = EVAP(K_evap,GLCe,V)

C_gln = EVAP(K_evap,GLNe,V)

C_glt = EVAP(K_evap,GLTe,V)

C_lac = EVAP(K_evap,LACe,V)

T_glc = TMM(Vm_t_glc,Km_t_glc,GLCe,GLC)

T_gln = TMM(Vm_t_gln,Km_t_gln,GLN,GLNe)

T_glt = TMM(Vm_t_glt,Km_t_glt,GLT,GLTe)

T_lac = TMM(Vm_t_lac,Km_t_lac,LAC,LACe)

T_o2 = K_t_o2*DIFF(O2e,O2)

T_o2e = 1-PULSE(A_p_o2,time,T_p_on_o2,F_p_on_o2,P_p_on_o2,T_p_off_o2,F_p_off_o2,P_p_off_o2)

V_ak = RMM(Vm_ak_f,Km_ak_atp,ATP,Km_ak_amp,AMP,0,1,Vm_ak_r,Km_ak_adp,ADP,0,1,0,1)

V_atpase = Vm_atpase*MM(Km_atpase_atp,ATP)*PULSE(A_p_atpase,time,T_p_on_atpase,F_p_on_atpase,P_p_on_atpase,T_p_off_atpase,F_p_off_atpase,P_p_off_atpase)

V_cdh = Vm_cdh*MM(Km_cdh_cit,CIT)*MM(Km_cdh_nad,NAD)

V_ck = RMM(Vm_ck_f,Km_ck_cr,Cr,Km_ck_atp,ATP,0,1,Vm_ck_r,Km_ck_pcr,PCr,Km_ck_adp,ADP,0,1)

V_cs = Vm_cs*MM(Km_cs_aca,ACA)*MM(Km_cs_oaa,OAA)

V_destress = Vm_destress*MM(Km_destress_anps,ANPs)

V_evap = K_evap

V_fai = RMM(Vm_fai_f,Km_fai_fbp,FBP,0,1,0,1,Vm_fai_r,Km_fai_g3p,G3P,0,1,0,1)

V_fbp = Vm_fbp*MM(Km_fbp_fbp,FBP)

V_fh = Vm_fh*MM(Km_fh_fum,FUM)

V_g6d = Vm_g6d*MM(Km_g6d_g6p,G6P)*MM(Km_g6d_nadp,NADP)

V_gns = RMM(Vm_gns_f,Km_gns_glt,GLT,0,1,0,1,Vm_gns_r,Km_gns_gln,GLN,0,1,0,1)

V_gtd = RMM(Vm_gtd_f,Km_gtd_glt,GLT,Km_gtd_nad,NAD,0,1,Vm_gtd_r,Km_gtd_akg,AKG,Km_gtd_nadh,NADH,0,1)

V_gyp = Vm_gyp*MM(Km_gyp_gly,GLY)

V_gys = Vm_gys*MM(Km_gys_g6p,G6P)*MM(Km_gys_atp,ATP)*SWITCH_I(GLY,TRH_i_gys_gly,F_i_gys_gly)

V_hk = Vm_hk*MM(Km_hk_glc,GLC)*MM(Km_hk_atp,ATP)*SWITCH_I(G6P,TRH_i_hk_g6p,F_i_hk_g6p)

V_iso = RMM(Vm_iso_f,Km_iso_g6p,G6P,0,1,0,1,Vm_iso_r,Km_iso_f6p,F6P,0,1,0,1)

V_kdh = Vm_kdh*MM(Km_kdh_akg,AKG)*MM(Km_kdh_adp,ADP)*MM(Km_kdh_nad,NAD)

V_ldh = RMM(Vm_ldh_f,Km_ldh_lac,LAC,Km_ldh_nad,NAD,0,1,Vm_ldh_r,Km_ldh_pyr,PYR,Km_ldh_nadh,NADH,0,1)

V_leak = Vm_leak*MM(Km_leak_nadh,NADH)*PULSE(A_p_leak,time,T_p_on_leak,F_p_on_leak,P_p_on_leak,T_p_off_leak,F_p_off_leak,P_p_off_leak)

V_mdh = Vm_mdh*MM(Km_mdh_mal,MAL)*MM(Km_mdh_nad,NAD)

V_op = Vm_op*MM(Km_op_nadh,NADH)*MM(Km_op_adp,ADP)*MM(Km_op_o2,O2)*RATIO(ADP,ATP)

V_os = V_op*(1-Eta_op)*PULSE(A_p_os,time,T_p_on_os,F_p_on_os,P_p_on_os,T_p_off_os,F_p_off_os,P_p_off_os)

V_pc = Vm_pc*MM(Km_pc_pyr,PYR)*MM(Km_pc_atp,ATP)

V_pdh = Vm_pdh*MM(Km_pdh_pyr,PYR)*MM(Km_pdh_nad,NAD)*MM(Km_pdh_coa,CoA)

V_pfk = Vm_pfk*MM(Km_pfk_f6p,F6P)*MM(Km_pfk_atp,ATP)*HILL(Ki_pfk_atp,ATP,nH_pfk)

V_pgk = Vm_pgk*MM(Km_pgk_g3p,G3P)*MM(Km_pgk_adp,ADP)

V_pk = Vm_pk*MM(Km_pk_pep,PEP)*MM(Km_pk_adp,ADP)

V_ppp = Vm_ppp*MM(Km_ppp_r5p,R5P)

V_sdh = Vm_sdh*MM(Km_sdh_suc,SUC)*MM(Km_sdh_nad,NAD)

V_stress = Vm_stress*MM(Km_stress_atp,ATP)*(1-SWITCH_I(ATP,TRH_i_stress_atp,F_i_stress_atp))*(-1+PULSE(A_p_leak,time,T_p_on_leak,F_p_on_leak,P_p_on_leak,T_p_off_leak,F_p_off_leak,P_p_off_leak))

********** MODEL REACTIONS

********** MODEL FUNCTIONS

MM(K,S) = (S/(K+S))

TMM(Vm,Km,Si,Se) = (Vm*(Si/(Si+Km)-Se/(Se+Km)))

RMM(Vmf,Kmf1,Sf1,Kmf2,Sf2,Kmf3,Sf3,Vmr,Kmr1,Sr1,Kmr2,Sr2,Kmr3,Sr3) = (Vmf*(Sf1/(Sf1+Kmf1))*(Sf2/(Sf2+Kmf2))*(Sf3/(Sf3+Kmf3))-Vmr*(Sr1/(Sr1+Kmr1))*(Sr2/(Sr2+Kmr2))*(Sr3/(Sr3+Kmr3)))

RMA(Kf,Sf1,Sf2,Sf3,Kr,Sr1,Sr2,Sr3) = (Kf*Sf1*Sf2*Sf3-Kr*Sr1*Sr2*Sr3)

DIFF(Se,Si) = (Se-Si)

PMM(Km,S,n) = (S^n/(Km^n+S^n))

EVAP(K_evap,Se,V) = (K_evap*Se/V)

RATIO(UPPER,LOWER) = (UPPER/LOWER)

HILL(Ki,S,n) = (1/(1+(S/Ki)^n))

SWITCH_I(S,St,a) = (1-1/(1+exp(-a*(S-St))))

SWITCH_A(S,St,a) = (1/(1+exp(-a*(S-St))))

PULSE(A,t,t1,a1,p1,t2,a2,p2) = ((1+unitpulseSB(time,t1,t2)*A*(1/(1+((1-p1)/p1)*exp(-a1*(t-t1)))-1/(1+((1-p2)/p2)*exp(-a2*(t-t2))))))

********** MODEL EVENTS

********** MODEL MATLAB FUNCTIONS
